# New ruthenium-xanthoxylin complex eliminates colorectal cancer stem cells by targeting the heat shock protein 90 chaperone

**DOI:** 10.1038/s41419-023-06330-w

**Published:** 2023-12-15

**Authors:** Luciano de S. Santos, Valdenizia R. Silva, Maria V. L. de Castro, Rosane B. Dias, Ludmila de F. Valverde, Clarissa A. G. Rocha, Milena B. P. Soares, Claudio A. Quadros, Edjane R. dos Santos, Regina M. M. Oliveira, Rose M. Carlos, Paulo C. L. Nogueira, Daniel P. Bezerra

**Affiliations:** 1 Gonçalo Moniz Institute, Oswaldo Cruz Foundation (IGM-FIOCRUZ/BA), Salvador, BA 40296-710 Brazil; 2 Department of Propedeutics, School of Dentistry of the Federal University of Bahia, Salvador, BA 40110-909 Brazil; 3 SENAI Institute of Innovation (ISI) in Health Advanced Systems, University Center SENAI/CIMATEC, Salvador, BA 41650-010 Brazil; 4 São Rafael Hospital, Rede D’Or/São Luiz, Salvador, BA 41253-190 Brazil; 5 Bahia State University, Salvador, BA 41150-000 Brazil; 6 Institute of Natural, Human and Social Sciences, Federal University of Mato Grosso, Sinop, MT 78557-267 Brazil; 7 Coordination of Science and Technology, Balsas Science Center, Federal University of Maranhão, Balsas, MA 65800-000 Brazil; 8 Department of Chemistry, Federal University of São Carlos, São Carlos, SP 13561-901 Brazil; 9 Department of Chemistry, Federal University of Sergipe, São Cristóvão, SE 49100-000 Brazil

**Keywords:** Cancer stem cells, Pharmacology

## Abstract

In this work, we describe a novel ruthenium-xanthoxylin complex, [Ru(phen)_2_(xant)](PF_6_) (RXC), that can eliminate colorectal cancer (CRC) stem cells by targeting the chaperone Hsp90. RXC exhibits potent cytotoxicity in cancer cell lines and primary cancer cells, causing apoptosis in HCT116 CRC cells, as observed by cell morphology, YO-PRO-1/PI staining, internucleosomal DNA fragmentation, mitochondrial depolarization, and PARP cleavage (Asp214). Additionally, RXC can downregulate the *HSP90AA1* and *HSP90B1* genes and the expression of HSP90 protein, as well as the expression levels of its downstream/client elements Akt1, Akt (pS473), mTOR (pS2448), 4EBP1 (pT36/pT45), GSK-3β (pS9), and NF-κB p65 (pS529), implying that these molecular chaperones can be molecular targets for RXC. Moreover, this compound inhibited clonogenic survival, the percentage of the CRC stem cell subpopulation, and colonosphere formation, indicating that RXC can eliminate CRC stem cells. RXC reduced cell migration and invasion, decreased vimentin and increased E-cadherin expression, and induced an autophagic process that appeared to be cytoprotective, as autophagy inhibitors enhanced RXC-induced cell death. In vivo studies showed that RXC inhibits tumor progression and experimental metastasis in mice with CRC HCT116 cell xenografts. Taken together, these results highlight the potential of the ruthenium complex RXC in CRC therapy with the ability to eliminate CRC stem cells by targeting the chaperone Hsp90.

## Introduction

Colorectal cancer (CRC) is the third most common cancer worldwide and the second leading cause of death from cancer, according to Global Cancer Statistics (GLOBOCAN). In 2020, it was estimated that there were more than 1.9 million new cases of CRC and 930,000 deaths [1]. By 2040, the CRC burden is expected to rise to 3.2 million new cases and 1.6 million deaths [2]. To address the growing cancer burden, CRC drug discovery must investigate new therapeutic approaches that target key cancer features.

Emerging evidence indicates that a subpopulation of CRC cells with stem cell characteristics, called cancer stem cells (CSCs), exhibits hierarchical organization and contributes to treatment resistance and tumor recurrence. CRC stem cells also give rise to highly metastatic cells, making elimination of this subpopulation of cells a key point for successful anti-CRC therapy [3–6].

Heat shock protein 90 (Hsp90) is a molecular chaperone related to a number of signaling proteins that are mutant, chimeric, or overexpressed in cancer cells. Cancer cells have higher levels of Hsp90 protein expression than healthy cells, and Hsp90 client proteins have been identified in over 100 different proteins [7–12]. In particular, high expression of Hsp90 is associated with poor prognosis in CRC patients [13, 14], and several clinical trials with Hsp90 inhibitors in various types of cancer have been reported with promising results [15–18].

**Fig. 1:**
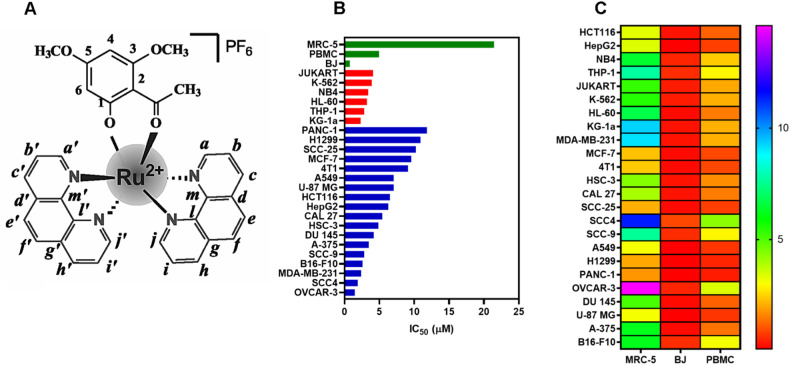
RXC displays cytotoxicity in solid and hematological cancers. **A** Chemical structure of RXC. **B** IC_50_ values in μM of RXC cytotoxicity to solid (blue bars) and hematological (red bars) cancers, as well as to noncancer cells (green bars). **C** Heatmap of selectivity indexes calculated for RXC.

A novel ruthenium-xanthoxylin complex (RXC, Fig. 1A), with the chemical formula *cis*-[Ru(phen)_2_(xant)](PF_6_) (where phen = 1,10′-phenanthroline and xant = xanthoxylin), was recently synthesized by our research group and showed potent cytotoxicity against HepG2 human hepatocellular carcinoma cells cultured in monolayers or in a 3D model of multicellular cancer spheroids. Mechanistically, RXC accumulated in cell nuclei, bound to DNA, inhibited DNA synthesis and arrested the cell cycle in S phase, leading to ERK1/2-mediated and p53-independent apoptosis in HepG2 cells. RXC also suppressed tumor growth in C. B-17 SCID mice engrafted with HepG2 cells [19]. In this work, we describe for the first time that RXC eliminates CRC stem cells by targeting the chaperone Hsp90.

## Results

### RXC exhibits potent cytotoxicity in cancer cell lines and primary cancer cells

RXC cytotoxicity was demonstrated against 24 cancer cell lines and three noncancer cells, as shown in Fig. 1B and Table S1, which display the IC_50_ values found. For the cancer cell lines, RXC had IC_50_ values ranging from 1.47 (OVCAR-3) to 11.83 (PANC-1) μM. When tested for cytotoxicity in noncancer cells, RXC had an IC_50_ value of 21.45 μM for MRC-5 cells, 0.76 μM for BJ cells and 4.93 μM for PBMCs. Figure 1C and Table S2 show the calculated selectivity indexes. The highest selectivity indexes were found for MRC-5 cells. Doxorubicin (DOX) had IC_50_ values ranging from 0.03 to 2.35 μM for JUKART and A549 cancer cells and 1.61, 1.28, and 0.73 μM for MRC-5, PBMC, and BJ noncancer cells, respectively.

We also cultured two histologic types of primary CRC, cholangiocarcinoma and papilliferous malignant mesothelioma cells, and treated them with 25 μg/ml RXC (31.3 μM) (Fig. S1). RXC treatment reduced cell viability by 79.8%, 37.4%, 79.2%, and 79.6%, respectively, whereas DOX at 25 μg/ml (46 μM) reduced cell viability by 71.0%, 36.2%, 65.6%, and 64.3%, respectively.

As RXC was cytotoxic to primary CRC, we decided to investigate its molecular mechanism of action in HCT116 CRC cells. Initially, RXC cytotoxicity was confirmed in HCT116 cells by trypan blue exclusion assay at concentrations of 2.5, 5 and 10 μM after 24, 48 and 72 h of incubation. RXC reduced the number of viable cells in a time- and concentration-dependent manner (Fig. S2).

### RXC causes apoptotic cell death in HCT116 CRC cells

**Fig. 2:**
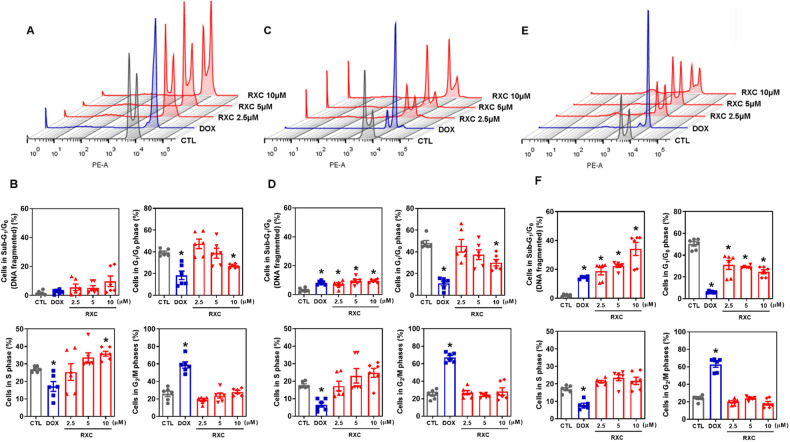
RXC induces cell cycle arrest in S-phase that is followed by DNA fragmentation. Effect of RXC on the cell cycle distribution of HCT116 cells after 24 (**A**, **B**), 48 (**C**, **D**) and 72 (**E**, **F**) h of incubation, as determined by flow cytometric analysis. The vehicle (0.2% DMSO) was used as a control (CTL), and doxorubicin (DOX, 1 µM) was used as a positive control. Data are shown as the mean ± S.E.M. of at least three repetitions (done in duplicate). *p < 0.05 compared to CTL by one-way ANOVA followed by Dunnett’s multiple comparisons test.

Cell cycle phases (G_0_/G_1_, S and G_2_/M) and internucleosomal DNA fragmentation were also quantified in RXC-treated HCT116 CRC cells (Fig. 2). All DNA that was smaller than a subdiploid (sub-G_0_/G_1_) was considered fragmented. At a concentration of 10 μM, RXC caused cell cycle arrest in S-phase after 24 h of incubation. A significant increase in cells with fragmented DNA was observed after 48 and 72 h of treatment with RXC. At concentrations of 2.5, 5, and 10 μM, RXC caused DNA fragmentation by 7.1%, 9.3% and 9.6% after 48 h of incubation (compared to 3.1% found in the control), whereas 18.8%, 22.4%, and 34.3% were found after 72 h of incubation (compared to 2.0% detected in the control), respectively. DOX induced G_2_/M arrest and increased the percentage of cells with fragmented DNA.

**Fig. 3:**
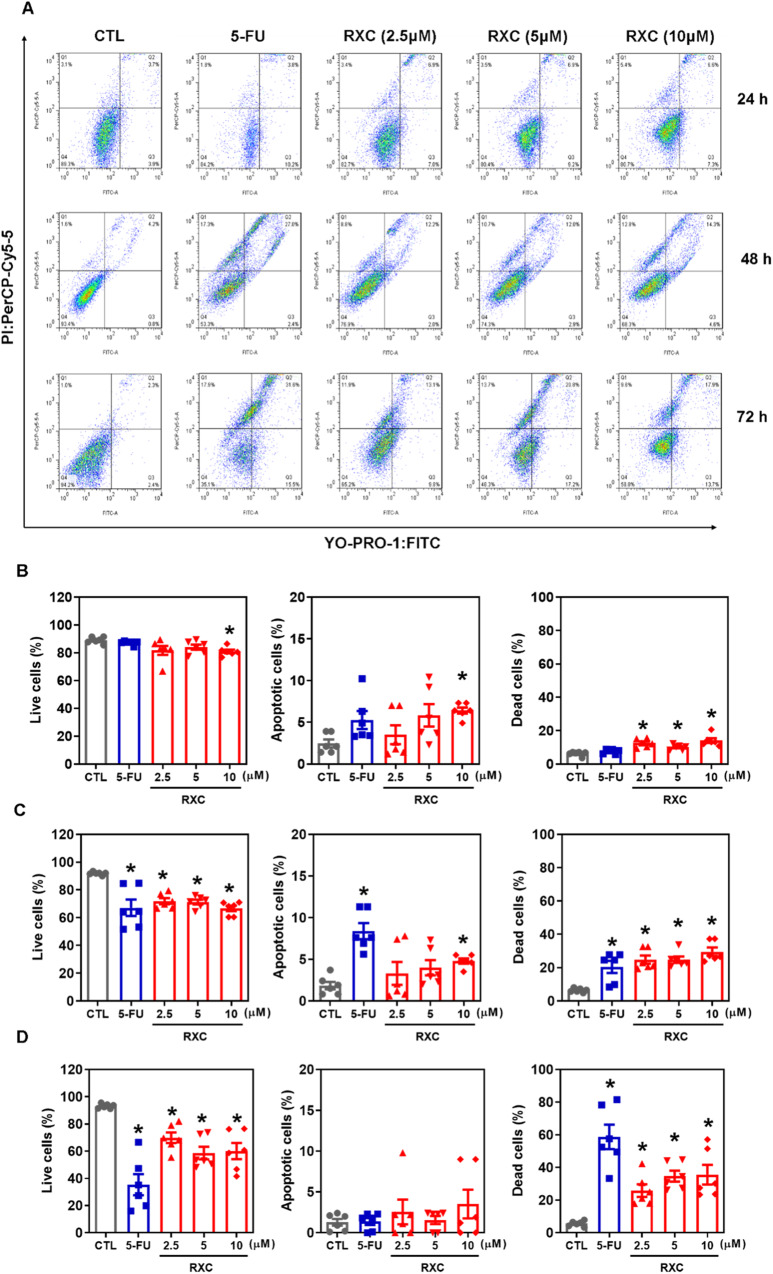
RXC causes apoptotic cell death. Effect of RXC on apoptosis induction in HCT116 cells after 24 (**A**, **B**), 48 (**A**, **C**) and 72 (**A**, **D**) h of treatment. The vehicle (0.2% DMSO) was used as a control (CTL), and 5-FU (23 µM) was used as a positive control. Data are shown as the mean ± S.E.M. of at least three repetitions (done in duplicate). *p < 0.05 compared to CTL by one-way ANOVA followed by Dunnett’s multiple comparisons test.

Cell viability was quantified using YO-PRO-1/propidium iodide (PI) staining in HCT116 CRC cells after incubation with RXC at concentrations of 2.5, 5, and 10 μM for 24, 48 and 72 h. The proportion of viable cells decreased, while the proportion of apoptotic and dead cells increased (Fig. 3). RXC also caused nuclear condensation after 24 h of incubation, as observed by the increase in side scatter, and cell shrinkage after 72 h of incubation, as measured by a reduction in forward light scatter. Both of these morphological changes are related to apoptotic cell death (Fig. S3).

**Fig. 4:**
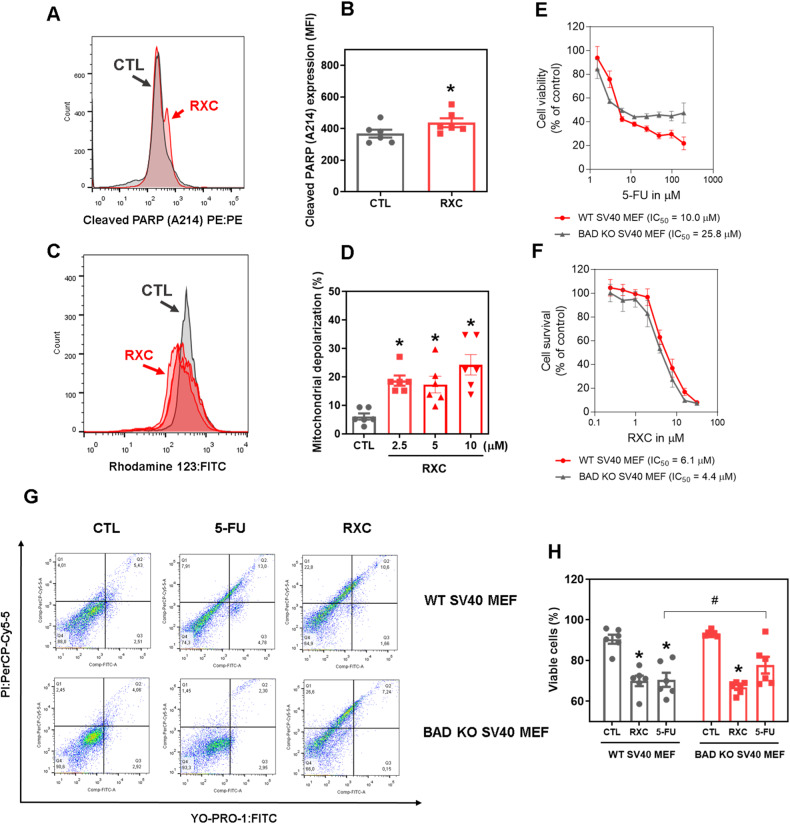
RXC induces mitochondria-mediated apoptosis. **A**, **B** Quantification of PARP-1 expression in HCT116 cells after 24 h of incubation with 10 μM RXC, as determined by flow cytometric analysis. *p < 0.05 compared to CTL by Student’s *t* test. **C**, **D** Quantification of mitochondrial membrane depolarization in HCT116 cells after 24 h of incubation, as determined by flow cytometry. *p < 0.05 compared to CTL by one-way ANOVA followed by Dunnett’s multiple comparisons test. **E**, **F** Survival curves of WT SV40 MEFs and BAD KO SV40 MEFs upon treatment with 5-FU and RXC. The curves were obtained from at least three repetitions (done in duplicate) using the Alamar blue assay after 72 h of incubation. **G**, **H** Induction of cell death in WT SV40 MEFs and BAD KO SV40 MEFs after 48 h of incubation with 40 μM 5-FU and 10 μM RXC. *p < 0.05 compared to CTL by one-way ANOVA followed by Dunnett’s multiple comparisons test. ^#^*p* < 0.05 compared to the respective treatment in the wild-type cell line by Student’s *t* test. The vehicle (0.2% DMSO) was used as a control (CTL). Data are shown as the mean ± S.E.M. of at least three repetitions (done in duplicate). MFI mean fluorescence intensity.

**Fig. 5:**
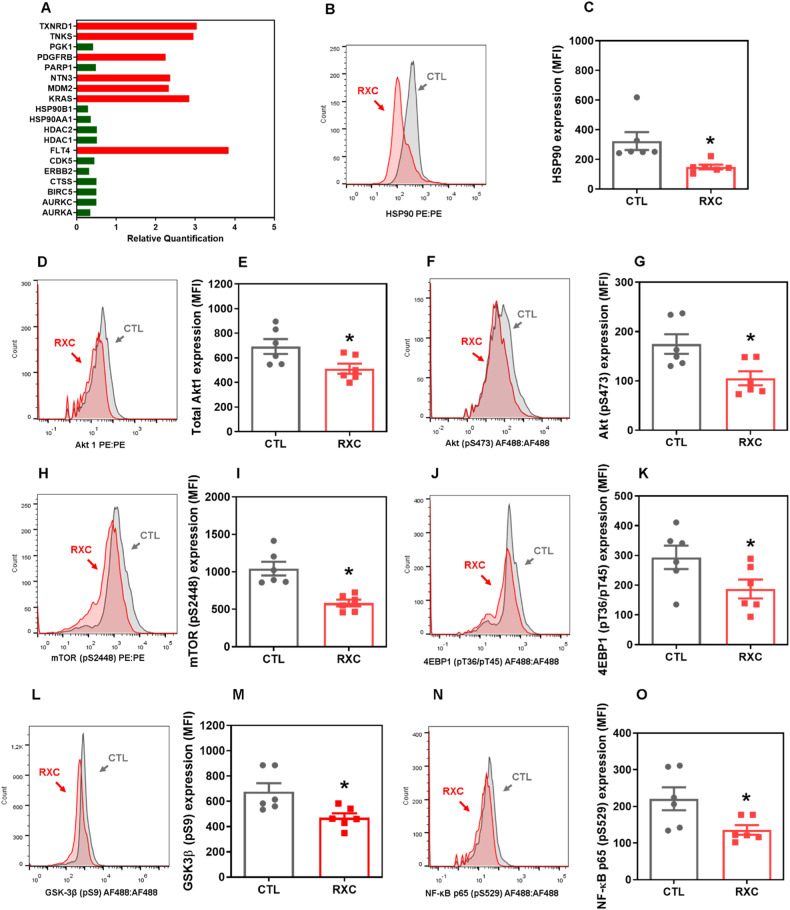
RXC targets the Hsp90 chaperone. **A** Genes up- and downregulated in HCT116 cells after 12 h of treatment with 10 µM RXC. The vehicle (0.2% DMSO) was used as a control (CTL). Data are shown as relative quantification (RQ) compared to CTL. The genes were considered to be upregulated if RQ ≥ 2 (red bars) and downregulated if RQ ≤ 0.5 (green bars). Quantification of Hsp90 (**B**, **C**), Akt1 (**D**, **E**), Akt (pS473) (**F**, **G**), mTOR (pS2448) (**H**, **I**), 4EBP1 (pT36/pT45) (**J**, **K**), GSK3β (pS9) (**L**, **M**), and NF-κB p65 (pS529) (**N**, **O**) expression in HCT116 cells after 24 h of incubation with 10 μM RXC, as determined by flow cytometric analysis. The vehicle (0.2% DMSO) was used as a control (CTL). Data are shown as the mean ± S.E.M. of at least three repetitions (done in duplicate). *p < 0.05 compared to CTL by Student’s *t* test. MFI mean fluorescence intensity.

Furthermore, we found increased levels of PARP cleavage (Asp214) (Fig. 4A, B) and decreased expression of the *BIRC5* and *CDK5* genes (Fig. 5A) in RXC-treated HCT116 CRC cells, indicating the occurrence of apoptotic cell death. The mitochondrial transmembrane potential was also determined in RXC-treated HCT116 CRC cells after 24 h of incubation. At all concentrations tested, RXC caused mitochondrial depolarization (Fig. 4C, D). Next, the BAD KO SV40 MEF cell line and its parental cell line WT SV40 MEF were used to assess the role of the proapoptotic protein BAD in RXC-induced cell death (Fig. 4E–H). On the other hand, RXC resulted in BAD-independent cell death.

### RXC targets the chaperone Hsp90 in HCT116 CRC cells

We discovered that RXC can downregulate the *HSP90AA1* (RQ = 0.35) and *HSP90B1* (RQ = 0.28) genes in CRC HCT116 cells by analyzing the transcripts of 82 target genes using a qPCR array, implying that disruption of these molecular chaperones could be a molecular target for RXC (Fig. 5A and Table S3). Therefore, we decided to quantify the levels of the Hsp90 protein and some of its downstream/client elements.

The expression of Hsp90 protein was decreased in RXC-treated cells (Fig. 5B, C), as well as the expression levels of Akt1 (Fig. 5D, E), Akt (pS473) (Fig. 5F, G), mTOR (pS2448) (Fig. 5H, I), 4EBP1 (pT36/pT45) (Fig. 5J, K), GSK-3β (pS9) (Fig. 5L, M), and NF-κB p65 (pS529) (Fig. 5N, O), and there was no change in the expression levels of Akt (pT308) (Fig. S4A, B), elF4E (pS209) (Fig. S4C, D), PI3K p85/p55 (pT458/pT199) (Fig. S4E,F), and S6 (pS235/pS236) (Fig. S4G, H). According to these findings, RXC appears to target the chaperone Hsp90 in HCT116 CRC cells.

GSK-3 is a Wnt signaling antagonist. In HCT116 CRC cells, RXC reduced the expression of GSK-3 (pS9), which is its inhibited form. As a result, we propose that RXC could inhibit Wnt signaling. On the other hand, cotreatment with lithium chloride, a Wnt activator, was ineffective in preventing RXC-induced cell death in HCT116 CRC cells (Fig. S5).

### RXC eliminates stem cells from HCT116 CRC cells

**Fig. 6:**
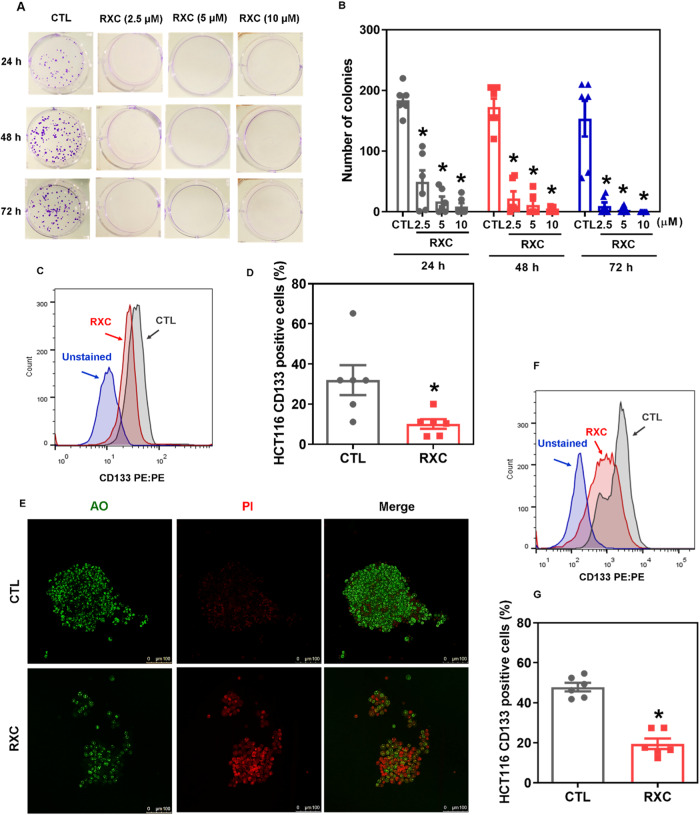
RXC suppresses CRC stem cells. **A** Representative images and (**B**) quantification of the number of colonies formed from HCT116 cells after treatment with RXC. *p < 0.05 compared to CTL by one-way ANOVA followed by Dunnett’s multiple comparisons test. **C**, **D** Quantification of CD133 expression on HCT116 cells after 24 h of incubation with 10 μM RXC, as determined by flow cytometric analysis. *p < 0.05 compared to CTL by Student’s *t* test. **E** Representative confocal images of colonospheres formed from HCT116 cells after 24 h of incubation with 10 μM RXC. Cells were stained with acridine orange (AO, green cells) and propidium iodide (PI, red cells that represent dead cells). **F**, **G** Quantification of CD133 expression in HCT116 cells cultured in colonospheres after 24 h of incubation with 10 μM RXC, as determined by flow cytometric analysis. *p < 0.05 compared to CTL by Student’s *t* test. The vehicle (0.2% DMSO) was used as a control (CTL). Data are shown as the mean ± S.E.M. of at least three repetitions (done in duplicate).

As Hsp90 has been linked to the properties of CSCs [20], we decided to evaluate the effect of RXC on CRC stem cells. By colony formation assay, we found that RXC can inhibit the clonogenic survival of HCT116 cells in a concentration- and time-dependent manner (Fig. 6A, B). Furthermore, the percentage of the CRC stem cell subpopulation was reduced by RXC, as observed by the reduction in CD133-positive cells, a CRC stem cell marker [21]. RXC-treated cells presented 10.1% HCT116 CD133-positive cells against 32.0% found in the control (Fig. 6C, D).

Furthermore, RXC reduced colonosphere formation in a concentration- and time-dependent manner (Fig. S6), indicating that RXC can eliminate CRC stem cells. In addition, dead cells were increased in colonospheres treated with RXC (Fig. 6E). The percentage of HCT116 CD133+ cells in RXC-treated colonospheres was also reduced. RXC-treated cells presented 19.5% HCT116 CD133-positive cells against 47.8% found in the control (Fig. 6F, G).

### RXC reduces the migration and invasion of HCT116 CRC cells

**Fig. 7:**
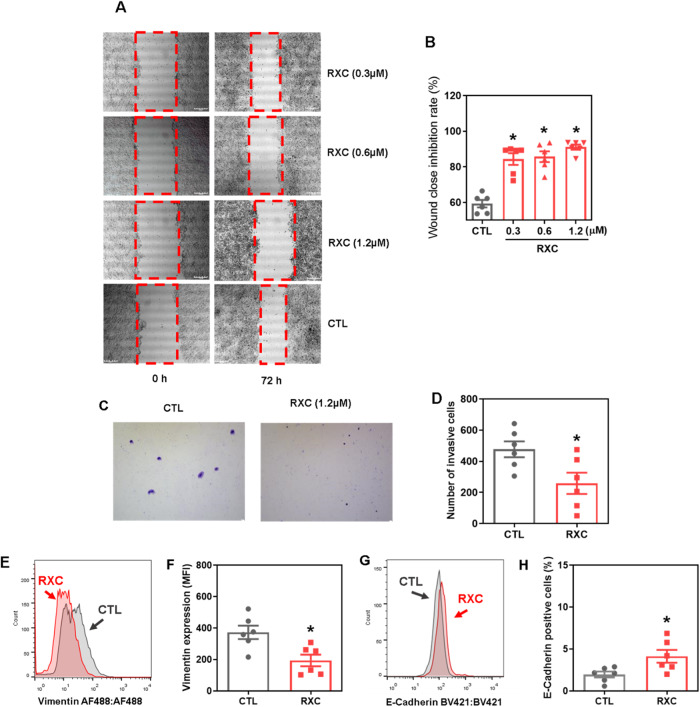
RXC reduces cell motility. **A** Representative images and (**B**) quantification of HCT116 cell migration in the wound healing assay after 72 h of incubation with RXC. *p < 0.05 compared to CTL by one-way ANOVA followed by Dunnett’s multiple comparisons test. **C** Representative images and (**D**) quantification of HCT116 cell invasion in the Transwell invasion assay after 48 h of incubation with 1.2 μM RXC. *p < 0.05 compared to CTL by Student’s *t* test. Quantification of (**E**, **F**) vimentin and E-cadherin (**G**, **H**) expression in HCT116 cells after 24 h of incubation with 10 μM RXC, as determined by flow cytometric analysis. *p < 0.05 compared to CTL by Student’s *t* test. The vehicle (0.2% DMSO) was used as a control (CTL). Data are shown as the mean ± S.E.M. of at least three repetitions (done in duplicate). MFI mean fluorescence intensity.

Cell motility is a hallmark of cellular malignancy and has been linked to the characteristics of CSCs [22]. As RXC can reduce the frequency of CRC stem cells, we decided to evaluate whether this molecule could reduce cell motility. Interestingly, RXC inhibited cell migration in HCT116 CRC cells in the wound healing assay (Fig. 7A, B) at noncytotoxic concentrations (Fig. S7).

Likewise, RXC inhibited cell invasion in the Transwell cell invasion assay using chambers coated with Matrigel to simulate the extracellular matrix (Fig. 7C, D). Then, the protein expression levels of the epithelial–mesenchymal transition (EMT) markers vimentin (Fig. 7E, F) and E-cadherin (Fig. 7G, H) were measured in CRC HCT116 cells treated with RXC. In RXC-treated cells, vimentin expression decreased and E-cadherin expression increased, indicating that RXC interferes with EMT in CRC HCT116 cells.

### RXC induces autophagy in HCT116 CRC cells

**Fig. 8:**
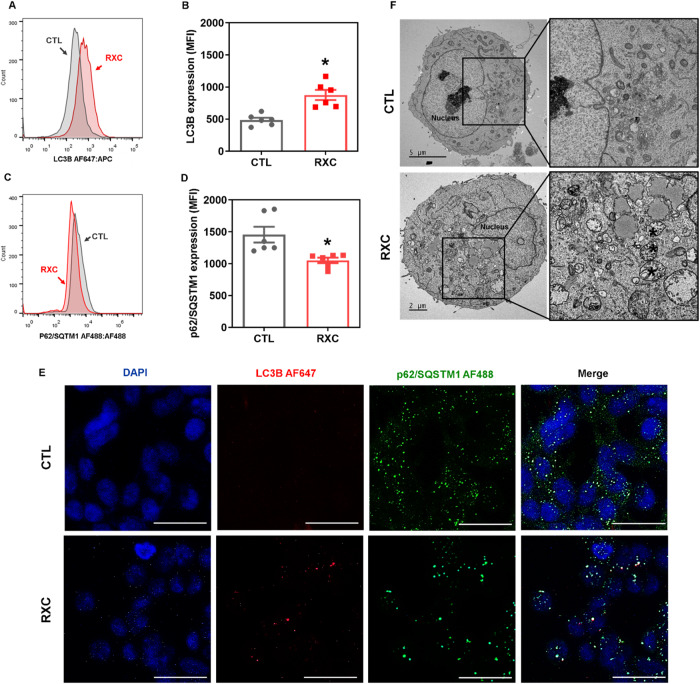
RXC induces autophagic process. Quantification of LC3B (**A**, **B**) and p62/SQSTM1 (**C**, **D**) expression in HCT116 cells after 24 h of incubation with 10 μM RXC, as determined by flow cytometric analysis. The vehicle (0.2% DMSO) was used as a control (CTL). Data are shown as the mean ± S.E.M. of at least three repetitions (done in duplicate). *p < 0.05 compared to CTL by Student’s *t* test. MFI mean fluorescence intensity. **E** Representative immunofluorescence images of LC3B and p62/SQSTM1 in HCT116 cells after 24 h of incubation with 10 μM RXC. Scale bar = 25 μm. **F** Representative MET images of HCT116 cells after 24 h of incubation with 10 μM RXC. Asterisks represent autophagic vacuoles. Scale bar = 2 or 5 μm.

We hypothesized that RXC could induce autophagy because it reduced the expression of mTOR, an autophagy inhibitor [23], in HCT116 CRC cells. We examined the levels of the autophagic markers LC3B and p62/SQSTM1 in RXC-treated HCT116 CRC cells to confirm this hypothesis. Surprisingly, RXC increased LC3B expression (Fig. 8A, B, E) while decreasing p62/SQSTM1 expression (Fig. 8C–E), indicating that RXC induces autophagy. Likewise, autophagic vacuoles were detected in RXC-treated HCT116 cells by transmission electron microscopy (TEM) analysis (Fig. 8F).

Since the autophagic process can have a cytoprotective (autophagy-related cell resistance) or cytotoxic (autophagy-mediated cell death) effect, we performed a functional cell death assay using two autophagic inhibitors (Fig. S8). We used 3-methyladenine (3-MA), an early-stage autophagy inhibitor that inhibits PI3K to prevent autophagosome formation [24], and chloroquine (CQ), a lysosomotropic agent that prevents autophagosome-lysosome fusion in late-stage autophagy [24]. The RXC-induced autophagic process appears to be cytoprotective, as both 3-MA and CQ enhanced RXC-induced cell death (Figs. S9 and S10).

### RXC inhibits tumor progression and experimental metastasis in mice with CRC HCT116 cell xenografts

**Fig. 9:**
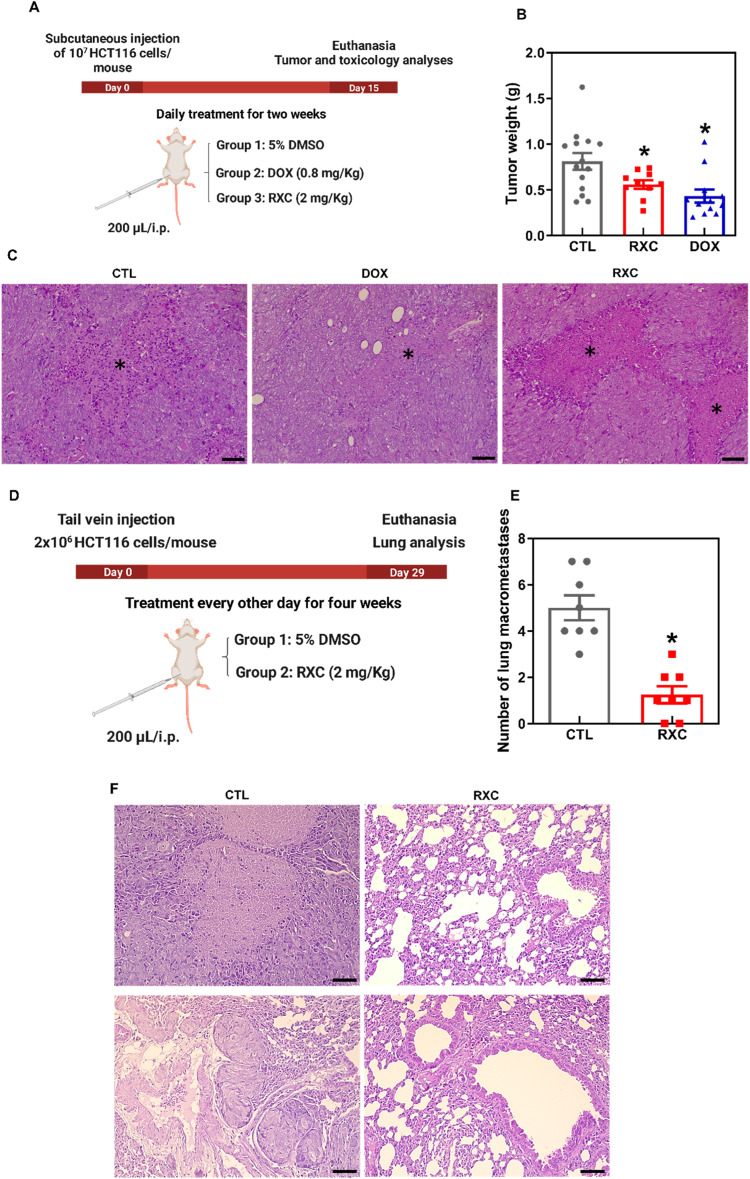
CXR exhibits antitumor potential in mouse models. **A** In vivo antitumor experimental design of RXC in C. B-17 SCID mice inoculated with HCT116 cells by subcutaneous injection. The animals were treated with RXC at a dosage of 2 mg/kg intraperitoneally once a day for 2 weeks. **B** In vivo antitumor activity of RXC. The vehicle (5% DMSO) was used as a control (CTL). DOX (0.8 mg/kg) was used as a positive control. Data are presented as the mean ± S.E.M. from 11–15 animals. *p < 0.05 compared to CTL by one-way ANOVA followed by Dunnett’s multiple comparisons test. **C** Representative histological analysis of HCT116 tumor tissues stained with hematoxylin and eosin and analyzed by light microscopy. The asterisks indicate areas of tissue necrosis. Scale bar = 50 μm. **D** In vivo antimetastatic experimental design of RXC in C. B-17 SCID mice grafted with HCT116 cells by tail vein injection. The animals were treated with 2 mg/kg RXC intraperitoneally every other day for 4 weeks. **E** The in vivo antimetastatic potential of RXC. The vehicle (5% DMSO) was used as a control (CTL). Data are presented as the mean ± S.E.M. from 8 animals. *p < 0.05 compared to CTL by Student’s *t* test. **F** Representative histological analysis of lung metastases stained with hematoxylin and eosin and analyzed by light microscopy. Scale bar = 50 μm.

The in vivo antitumor activity of RXC was studied in C. B-17 SCID mice subcutaneously inoculated with HCT116 CRC cells. The animals were treated intraperitoneally with 2 mg/kg RXC once a day for 2 weeks (Fig. 9A). At the end of treatment, the mean weight of tumors in control animals was 0.81 ± 0.09 g, while it was 0.6 ± 0.05 g in RXC-treated animals (Fig. 9B). This represents a tumor mass inhibition of 30.9% by RXC, whereas DOX reduced the tumor mass by 46.7%. Areas of coagulative necrosis and inflammatory cells were found more extensively in tumor tissues from animals treated with RXC and DOX (Fig. 9C).

Toxicological parameters were also assessed in animals given RXC. Animals given RXC and DOX had a decrease in body weight (*p* < 0.05) (Fig. S11), and four animals of each treated group died during the treatment. No significant changes in organ weights were observed in any group. In the hematological analysis, a reduction in erythrocytes was found in RXC-treated animals. Decreases in erythrocytes, hemoglobin, hematocrit, MCV and leukocytes were observed in DOX-treated animals (Fig. S12).

The renal architecture was partially preserved, mainly due to the reduction in Bowman’s space caused by glomerular hyalinization (moderate to intense) and by the focal areas of coagulation necrosis of the renal cortex tubules, which was more evident in the animals treated with DOX and RXC. Furthermore, moderate to intense vascular hyperemia was observed in the kidneys of all groups (Fig. S13).

The animals had partially preserved hepatic architecture due to hydropic degeneration (moderate to severe) and focal areas of hepatocyte coagulation necrosis, mainly in animals treated with DOX and RXC (Fig. S13). In addition, a DOX-treated animal showed intense microvacuolar fatty degeneration. Mild to moderate vascular hyperemia of the sinusoids and centrilobular veins was observed in all experimental groups. The portal architecture ranged from preserved to partially preserved, highlighting a dilation of the hepatic artery, vascular hyperemia and punctual accumulation of mononuclear cells.

The architecture of the lung parenchyma was partially preserved in all animals in the present study, mainly due to the decrease in air space (atelectasis) due to hyperplasia/hypertrophy of the alveolar septa (Fig. S13). The histopathological changes observed were vasodilation, vascular hyperemia and edema, which ranged from mild to severe. In addition, focal areas of fibrosis and inflammation were observed in all experimental groups. Furthermore, an important area of hemorrhage with hemosiderin deposits was observed in one animal from the RXC group. No histological changes were observed in the hearts of animals in any of the experimental groups.

The antimetastatic potential of RXC was investigated in C. B-17 SCID mice inoculated intravenously with HCT116 cells via tail vein injection. For 4 weeks, the animals received 2 mg/kg RXC intraperitoneally every other day (Fig. 9D). The mean number of pulmonary metastatic nodules in CXR-treated animals was 1.3 ± 0.4 compared to 5.0 ± 0.5 in the control group (Fig. 9E). The histological analysis of the lungs demonstrated the presence of metastatic nodules only in the animals of the control group. The nodules were of various sizes, and the more extensive nodules had necrotic centers (Fig. 9F).

## Discussion

**Fig. 10:**
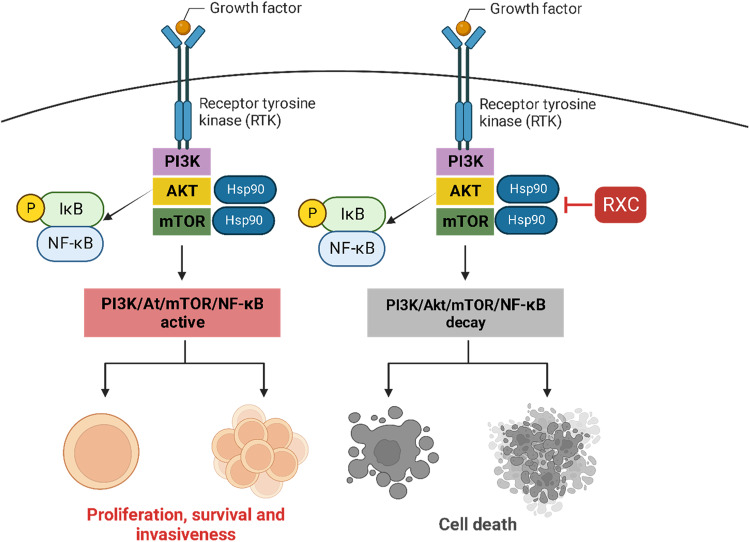
Proposed molecular mechanism of action for RXC. Inhibition of Hsp90 is a central target of the mechanism of action of RXC, which leads to the suppression of PI3K/Akt/mTOR/NF-κB signaling and causes cell death.

In this study, we demonstrate for the first time that RXC targets the molecular chaperone Hsp90 as a mechanism of cytotoxicity (Fig. 10). RXC showed strong cytotoxicity to cancer cell lines and primary cancer cells of various histological types. RXC eliminated CRC stem cells and induced apoptosis in HCT116 CRC cells along with Hsp90 inhibition. CRC HCT116 cells treated with RXC also induced cytoprotective autophagy and inhibited cell migration and invasion. RXC inhibited the progression of CRC HCT116 cells in vivo and experimental lung metastases in xenograft models.

As mentioned above, RXC was previously described as a cytotoxic agent against cancer cells with the ability to cause p53-independent and ERK1/2-mediated apoptotic cell death in liver cancer cells [19]. Herein, we demonstrated that RXC is cytotoxic to cancer cell lines and primary cancer cells, leading CRC HCT116 cells to undergo apoptosis. Curiously, different ruthenium complexes have been reported to induce apoptotic cell death in cancer cells [25–29].

RXC also eliminated CRC stem cells and inhibited the Hsp90 chaperone. Furthermore, the Hsp90 downstream/client elements Akt1, Akt (pS473), mTOR (pS2448), 4EBP1 (pT36/pT45), GSK-3β (pS9) and NF-κB p65 (pS529) were also suppressed in RXC-treated cells. Carvalho et al. [19] previously reported that RXC accumulates in cell nuclei and binds to DNA. This suggests that the ability of RXC to target the Hsp90 chaperone may be due to its role in interacting with cellular DNA. Interestingly, both the gene and protein expression levels of Hsp90 were affected by RXC treatment in CRC cells.

Hsp90 is needed for the folding and stabilization of many signaling oncoproteins. Interestingly, this chaperone has been reported to facilitate stemness in cancer cells [10, 20, 30, 31]. In particular, the CSC markers CD133 and Sox2 were upregulated in U87MG and U251 glioma cells stably overexpressing HSP90 (oe-HSP90) and downregulated after HSP90 knockdown (sh-HSP90) [20]. Likewise, mammospheres, which are enriched breast stem/progenitor populations, show higher Hsp90 expression than cells grown in monolayer cultures [31].

This is the first report on a ruthenium complex with the ability to inhibit the Hsp90 chaperone. In addition to Hsp90 inhibition, RXC also reduced the expression of the *AURKC*, *AURKA*, *CTSS* and *ERBB2* genes, which may also contribute to its cytotoxicity. A Ru(II) triazine complex was reported to inhibit CSCs in MCF-7 CD44-positive cells and HCT-116 CD44-positive cells by suppressing GRP-78 levels [32].

RXC not only reduced the CRC stem cell subpopulation but also reduced cell migration and invasion in HCT116 CRC cells. CSCs play a critical role in the metastasis and recurrence of CRC, and increased cell motility is a key feature of metastatic cancer cells [22, 33, 34]. Polypyridyl Ru(II) complexes have also been reported to reduce migration and invasion in melanoma and breast cancer cells [35]. A complex of organoruthenium with 8-hydroxyquinoline also inhibited migration and invasion in bone, lung, and breast cancer cells [36].

Cell motility and metastasis are associated with EMT [37]. In particular, the EMT markers vimentin and E-cadherin were modulated by RXC treatment. Akt/mTOR/NF-κB signaling activation has been reported to phosphorylate and activate EMT transcription factors [38–40]. Importantly, the key molecules of these cell signaling pathways are downstream/client elements of the Hsp90 chaperone and were suppressed by RXC treatment, indicating that the effects of RXC on cell motility may be due to suppression of Akt/mTOR/NF-κB signaling.

Mechanistic target of rapamycin (mTOR) initiates the formation of two different complexes, mTORC1 and mTORC2. mTORC1 inhibits the earliest phases of autophagy by blocking the phosphorylation-dependent Atg13 and ULK1 proteins and limiting the cell’s degradative capability by reducing the activity of TFEB family members. mTORC2 also regulates autophagic flow and lysosomal capacity via transcription-independent and transcription-dependent processes [23, 41, 42]. mTORC1 contains mTOR phosphorylated predominantly at S2448 [43]. Interestingly, RXC downregulated mTOR (pS2448) expression in HCT116 CRC cells, indicating that RXC’s capacity to induce autophagy may be attributable to its function in suppressing mTOR phosphorylation.

Autophagy is an intracellular degradation process that occurs under various stressful conditions, including organelle damage, the presence of abnormal proteins, and nutrient deprivation. In cancers, autophagy modulation performs oncogenic or tumor inhibitory functions during tumor development or cytoprotective (chemoresistance) or cell damage (autophagy-mediated cell death) functions during cancer treatment [24, 44, 45]. In particular, the Nomenclature Committee on Cell Death (NCCD) considers autophagic cell death only in cases where cell death can be reversed by genetic or pharmacological inhibition of the autophagic machinery [46]. RXC-induced autophagy in CRC HCT116 cells appears to have cytoprotective functions, since CQ and 3-MA autophagy inhibitors increased its cytotoxicity. Cytoprotective autophagy is often observed with mTOR inhibitor therapies. Some autophagy inhibitors have been used in combination with these drugs to prevent resistance and improve cell sensitivity [24, 47, 48].

The in vivo antitumor activity of RXC was assessed in C. B-17 SCID mice grafted subcutaneously with HCT116 cells. RXC treatment reduced HCT116 cell growth by 30.9%. Furthermore, RXC also reduced experimental lung metastases in SCID C. B-17 mice grafted intravenously through the tail vein with HCT116 cells, indicating antitumor and antimetastatic potential. Previously, RXC was reported to inhibit the in vivo growth of HepG2 cells [19].

Two Ru(II) complexes with piplartine and one Ru(II)-thymine complex also reduced tumor growth in HCT116-grafted mice [49, 50]. A Ru(II) triazine complex also reduced HCT-116 CD133-positive cell development in xenograft mice [32]. A study showed that a Ru(II) complex with terpyridine inhibited tumor growth in mice inoculated with a mouse colon carcinoma cell line (CT26) [51]. A complex of Ru(II) with 6-methyl-2-thiouracil also inhibited the development of HL-60 cells in vivo [52]. Ru(II) complexes containing heterocyclic thioamidates have also been reported to inhibit the development of HepG2 cells in a xenograft model [53].

In this work, we showed that RXC had a potent cytotoxic effect on cancer cell lines and primary cancer cells. It caused apoptotic cell death and cytoprotective autophagy, and inhibited the chaperone Hsp90, CSCs and migration and invasiveness in HCT 116 CRC cells. Furthermore, RXC showed antitumor and antimetastatic potential in vivo. Taken together, these results highlight the potential of this compound in CRC therapy with the ability to eliminate CRC stem cells by targeting the chaperone Hsp90.

## Material and methods

### RXC synthesis

RXC was synthesized and characterized in the same manner as previously described [19]. For all experiments, RXC was dissolved in sterile dimethyl sulfoxide (DMSO, Synth, Diadema, SP, Brazil) in a 5 mg/ml stock solution and diluted with culture medium at various concentrations.

### Cell culture

This study used a panel of 24 cancer cell lines, four primary cancer cells, two noncancer cell lines, one primary noncancer cells, and one mutant cell line and its parental cells, as detailed in Table S4. The cells were cultured according to the manufacturer’s instructions for each cell line or the ATCC guidelines for animal cell culture. All cell lines were cultured in flasks at 37 °C in 5% CO_2_ and subcultured every 3–4 days to maintain exponential growth. A 0.25% trypsin EDTA solution was used to collect adherent cells (Sigma-Aldrich Co.). All cell lines were tested for mycoplasma using a mycoplasma stain kit to validate the use of mycoplasma-free cells (Sigma-Aldrich).

### In vitro studies

#### Trypan blue exclusion assay

The trypan blue exclusion assay was used to quantify the number of viable cells. Briefly, 90 μl of the cell suspension plus 10 μl of trypan blue (0.4%) were mixed, and an aliquot was analyzed in a hemocytometer. The number of viable (unstained cells) and nonviable (trypan blue-stained cells) cells was counted using a light microscope.

#### Alamar blue assay

The Alamar blue assay was used to determine cell viability as previously described [54]. Cells were seeded into 96-well plates at a density of 7 × 10^3^ cells per well for adherent cells or 3 × 10^4^ cells per well for suspended cells. The drug was added to each well after 24 h of incubation for adherent cells or immediately for suspended cells and incubated for an additional 72 h. The positive control included DOX (Laboratorio IMA S.A.I.C., Buenos Aires, Argentina) or 5-fluorouracil (5-FU, Sigma-Aldrich Co.). Each well received 100 µM resazurin (Sigma-Aldrich Co. St. Louis, MO, United States) four (for cell lines) or 24 (for primary culture) h before the end of the incubation. A SpectraMax 190 Microplate Reader (Molecular Devices, Sunnyvale, CA, USA) was used to measure absorbance at 570 and 600 nm.

#### Flow cytometry assays

For quantification of internucleosomal DNA fragmentation and cell cycle distribution, the cells were stained with PI using a solution containing 0.1% Triton X-100, 2 µg/ml PI, 0.1% sodium citrate, and 100 µg/ml RNAse (all from Sigma-Aldrich) and incubated in the dark for 15 min at room temperature [55]. Finally, cell fluorescence was measured using flow cytometry.

To ensure mitochondrial transmembrane potential, the cells were stained with rhodamine 123 (5 µg/ml, Sigma-Aldrich Co.) at 37 °C for 15 min in the dark [56]. The cells were then washed with saline and incubated in saline for 30 min in the dark at 37 °C, and cell fluorescence was determined using flow cytometry.

For apoptosis detection or functional assays, cell viability was quantified using annexin V-FITC/PI (FITC Annexin V Apoptosis Detection Kit I, BD Biosciences, San Jose, CA, USA) or YO-PRO-1/PI (Sigma-Aldrich Co.). The following inhibitors were used: chloroquine (autophagy inhibitor, Ipca Laboratories, Mumbai, MH, India); 3-methyladenine (autophagy inhibitor, Sigma-Aldrich Co.); and lithium chloride (Wnt activator, Sigma-Aldrich Co.).

Flow cytometry was used to measure protein expression levels using primary antibodies conjugated with specific fluorochromes, as detailed in Table S5. For intracellular protein staining, cells were collected and resuspended in 0.5–1 ml of 4% formaldehyde for 10 min at 37 °C. The tubes were then kept on ice for 1 min and permeabilized for 30 min on ice by slowly adding ice-cold 100% methanol to prechilled cells while gently vortexing to a final concentration of 90% methanol. After washing with incubation buffer (0.5% bovine serum albumin in PBS), antibodies were added and incubated at room temperature for 1 h. Finally, the cells were washed with PBS, and the fluorescence of the cells was measured using flow cytometry.

For cell surface protein staining, cells were washed with incubation buffer (0.5% bovine serum albumin in PBS), and then antibodies were added and incubated for 1 h at room temperature. The cells were then washed with PBS, and cell fluorescence was measured using flow cytometry. To select viable cells for quantification of CD133-positive cells, YO-PRO-1 (Sigma-Aldrich Co.) was used.

For all flow cytometry analyses, cell fluorescence was measured with a BD LSRFortessa cytometer and analyzed by BD FACSDiva Software (BD Biosciences) and FlowJo Software 10 (FlowJo LLC; Ashland, OR, USA). At least 10,000 events were evaluated per sample for intracellular staining, and at least 30,000 events were acquired per sample for cell surface protein staining. FSC-A versus FCS-H and SCC-A versus SCC-H were used to remove the doublets. The analysis excluded cellular debris.

### qPCR array

Total RNA was isolated using the RNeasy Plus Mini Kit (Qiagen; Hilden, Germany) according to the manufacturer’s instructions. The RNA was tested for purity and quantified using a NanoDrop® 1000 spectrophotometer (Thermo Fisher Scientific, Waltham, Massachusetts, USA). The Superscript VILOTM Kit was used for RNA reverse transcription (Invitrogen Corporation; Waltham, MA, USA). TaqMan® array human cancer drug targets 96-well plate, fast (ID RPRWENH, Applied BiosystemsTM, Foster City, CA, USA) was used for gene expression quantification by qPCR. The reactions were carried out in an ABI ViiA7 system (Applied BiosystemsTM).

The cycle conditions were 2 min at 50 °C, 10 min at 95 °C, and 40 cycles of 15 s at 95 °C and 1 min at 60 °C. All experiments were carried out in DNase/RNase-free environments. The 2^−ΔΔCT^ method [57] was used to calculate the relative quantification (RQ) of mRNA expression using Gene Expression SuiteTM Software (Applied BiosystemsTM), and cells treated with the negative control (0.2% DMSO) served as a calibrator. The RQs of the reference genes HPRT1, TFRC and YWHAZ were used to normalize the reactions.

The genes were considered upregulated if RQ ≥ 2, indicating that gene expression in RXC-treated cells was at least twice that of the negative control-treated cells. Similarly, genes were considered downregulated if RQ ≤ 0.5, indicating that gene expression in RXC-treated cells was at least half that of the negative control-treated cells.

### Immunofluorescence staining

The cells were plated onto coverslips in 24-well plates and treated for 24 h with the drug. Next, the cells were washed twice with saline solution, permeabilized with 0.5% Triton X-100, treated with RNAse (10 μg/ml), and incubated overnight with primary antibodies conjugated with a specific fluorochrome (see Table S5 for antibody details). The cells were washed with PBS the next day and mounted with Fluoromount-G with DAPI (Invitrogen, Thermo Fisher Scientific). A Leica TCS SP8 confocal microscope was used to examine the cells (Leica Microsystems, Wetzlar, HE, Germany).

### Colony-forming assay

To test clonogenic ability, 1000 cells were seeded in 6-well plates with 6 ml of complete medium and incubated with the drug for 24, 48, and 72 h. The medium was then changed to drug-free fresh medium and cultured for a total of 14 days. The cells were then fixed in methanol and stained with 0.5% crystal violet. The number of colonies with more than 50 cells was counted using an optical microscope (Nikon, TS100).

### Colonosphere assay

HCT116 cells, at densities of 1.25 × 10^5^ cells/ml, were cultured in 24-well low adhesion plates (Corning, USA) in serum-free DMEM-F12 supplemented with 20 ng/ml bFGF (PeproTech, USA), 20 ng/ml EGF (PeproTech, USA), and B27 supplement (Invitrogen, Carlsbad, CA, USA). On the following day, the cells were subjected to drug concentrations of 20, 10, 5, 2.5, and 1.25 μM. The cells were photographed using an optical microscope after 0, 24, 48, and 72 h of incubation (Leica, DMI8). Furthermore, 10 µM RXC-treated cells were stained with acridine orange/PI and analyzed by confocal microscopy or stained with anti-CD133 antibody plus YO-PRO-1 and analyzed by flow cytometry.

### Wound healing assay

Wound healing assays were carried out as previously described [58], with some modifications. Cells were grown to 80–90% confluency in 12-well plates, and a wound was created by dragging a plastic pipette tip across the cell surface. The remaining cells were washed three times in saline to remove cell debris before being incubated in serum-free medium and treated with the drug. Using an optical microscope, migrating cells in front of the wound were photographed after 0 and 72 h of incubation (Nikon, TS 100). ImageJ software was used to calculate the wound area (NIH, USA).

### Transwell invasion assay

Transwell plates were used for the cell invasion assay, as previously described [59]. Cells were first incubated in serum-free medium for 24 h. Cell culture inserts in 6-well plates (8 μm pore size; Corning, USA) precoated with Matrigel (Corning, USA) were used. In the upper chamber, 10^6^ cells were suspended in 1.5 ml of serum-free medium, while the lower chamber received 2 ml of medium containing 20% FBS. Cotton swabs were used to remove cells that remained in the upper chamber after 48 h of incubation. The cells on the lower surface of the membrane were fixed in 4% paraformaldehyde and stained with 0.5% crystal violet. Using an optical microscope, the cells were photographed and counted (Leica, DMI8).

### Transmission electron microscopy analyses

Initially, the cells were fixed in 100 μM sodium cacodylate buffer (pH 7.4) containing 2.5% glutaraldehyde and 2% paraformaldehyde for at least 2 h. After rinsing, the cells were treated with 1% osmium tetroxide, 0.8% potassium ferricyanide, and 5 mM calcium chloride for 1 h. After rinsing again, the cells were dehydrated in an acetone series and embedded in polybed epoxy resin. The ultrathin sections were stained with 2% aqueous uranyl acetate and 2% aqueous lead citrate before TEM analysis with a JEM-1230 microscope (JEOL, 1230, USA, Inc.).

### In vivo studies

#### Animals

A total of 61 C. B-17 SCID mice (female, 20–25 g) were supplied and housed under specific pathogen-free conditions by FIOCRUZ-BA animal facilities (Salvador, Bahia, Brazil) in accordance with an experimental protocol approved by a local animal ethics committee (#10/2020). All mice were fed a standard pellet diet (with free access to food and water) and kept in an artificially lit room (12 h dark/light cycle).

#### CRC tumor growth xenograft model

HCT116 cells (10^7^ cells/500 μl) were inoculated subcutaneously into the left front armpit of mice, as previously reported [49, 50]. The animals were then treated intraperitoneally (200 μl/animal) once a day for 2 weeks. The animals were divided into three groups based on randomization: group 1 received the vehicle (5% DMSO solution) (*n* = 15); group 2 received DOX at a dose of 0.8 mg/kg (*n* = 15); and group 3 received RXC at a dose of 2 mg/kg (*n* = 15). The animals were euthanized with an anesthetic overdose (thiopental, 100 mg/kg) 1 day after treatment ended, and tumors were excised, weighed, and processed for histological analysis. The inhibition ratio (percent) was calculated as follows: inhibition ratio (percent) = [(A − B)/A] × 100, where A is the average tumor weight of the negative control and B is the treated group.

#### CRC experimental lung metastasis xenograft model

HCT116 cells (2 × 10^6^ cells/100 μl) were injected into the tail vein of each mouse. The animals were then treated intraperitoneally (200 μl/animal) every other day for 4 weeks. The animals were randomized into two groups: group 1 received vehicle (5% DMSO solution) (*n* = 8), and group 2 received RXC at a dose of 2 mg/kg (*n* = 8). The animals were euthanized with an anesthetic overdose (thiopental, 100 mg/kg) 1 day after treatment ended, and their lungs were excised and fixed in 4% formaldehyde. The number of lung macrometastases per animal was counted and processed for histological analysis.

#### Toxicological aspects

To evaluate toxicological features, all animals were weighed at the beginning and end of the experiment. Throughout the experiment, the animals were monitored for abnormalities. An Advia 60 hematology system was used to perform hematological analyses (Bayer, Leverkusen, Germany). The livers, kidneys, lungs, and hearts were removed, weighed, and examined for color change, gross lesion formation, and/or hemorrhaging before being fixed in 4% formaldehyde, dehydrated in a graded alcohol series, washed in xylene, and embedded in paraffin wax. The tissue was cut into 5 μm thick slices, stained with hematoxylin-eosin and/or periodic acid-Schiff (liver and kidney), and examined histologically under optical microscopy.

### Statistical analysis

The data are presented as the mean ± S.E.M. or as IC_50_ values with a 95% confidence interval of at least three repetitions (done in duplicate). The two-tailed unpaired Student’s *t* test was used to compare data with two groups, and one-way analysis of variance (ANOVA) followed by Dunnett’s multiple comparisons test was used to compare data with three or more groups. All statistical analyses were performed with GraphPad Prism (Intuitive Software for Science; San Diego, CA, USA).

### Reporting summary

Further information on research design is available in the Nature Research Reporting Summary linked to this article.

### Supplementary information

Supplemental material

Reporting Summary

## Data Availability

The data in the current study are available from the corresponding authors upon reasonable request.
