# Supplementary material for: New ruthenium-xanthoxylin complex eliminates colorectal cancer stem cells by targeting the heat shock protein 90 chaperone
Source: Cell Death Dis. 2023 Dec 15;14(12):832. doi: 10.1038/s41419-023-06330-w (PMC10724293; doi:10.1038/s41419-023-06330-w)
Supplement: Supplementary file 1 — Supplemental material [file 41419_2023_6330_MOESM1_ESM.pdf]

Supplementary Material

New ruthenium-xanthoxylin complex eliminates colorectal cancer stem cells by targeting the heat shock protein 90 chaperone

Luciano de S. Santos[1], Valdenizia R. Silva[1], Maria V. L. de Castro[1], Rosane B. Dias[1,2], Ludmila de F. Valverde[1], Clarissa A. G. Rocha[1,2], Milena B. P. Soares[1,3], Claudio A. Quadros[4,5], Edjane R. dos Santos[6], Regina M. M. Oliveira[7], Rose M. Carlos[8], Paulo C. L. Nogueira[9], Daniel P. Bezerra[1,*]

[1]Gonçalo Moniz Institute, Oswaldo Cruz Foundation (IGM-FIOCRUZ/BA), Salvador, Bahia, 40296-710, Brazil.

[2]Department of Propedeutics, School of Dentistry of the Federal University of Bahia, Salvador, Bahia, 40110-909, Brazil.

[3]SENAI Institute of Innovation (ISI) in Health Advanced Systems, University Center SENAI/CIMATEC, Salvador, Bahia, 41650-010, Brazil.

[4]São Rafael Hospital, Rede D'Or/São Luiz, Salvador, Bahia, 41253-190, Brazil.

[5]Bahia State University, Salvador, Bahia, 41150-000, Brazil.

[6]Institute of Natural, Human and Social Sciences, Federal University of Mato Grosso, Sinop, MT, 78557-267, Brazil.

[7]Coordination of Science and Technology, Balsas Science Center, Federal University of Maranhão, Balsas, Maranhão, 65800-000, Brazil.

[8]Department of Chemistry, Federal University of São Carlos, São Carlos, São Paulo, 13561-901, Brazil.

[9]Department of Chemistry, Federal University of Sergipe, São Cristóvão, Sergipe, 49100-000, Brazil.

***Corresponding author:** D. P. Bezerra, E-mail: daniel.bezerra@fiocruz.br Tel/Fax + 55 71 3176 2272.

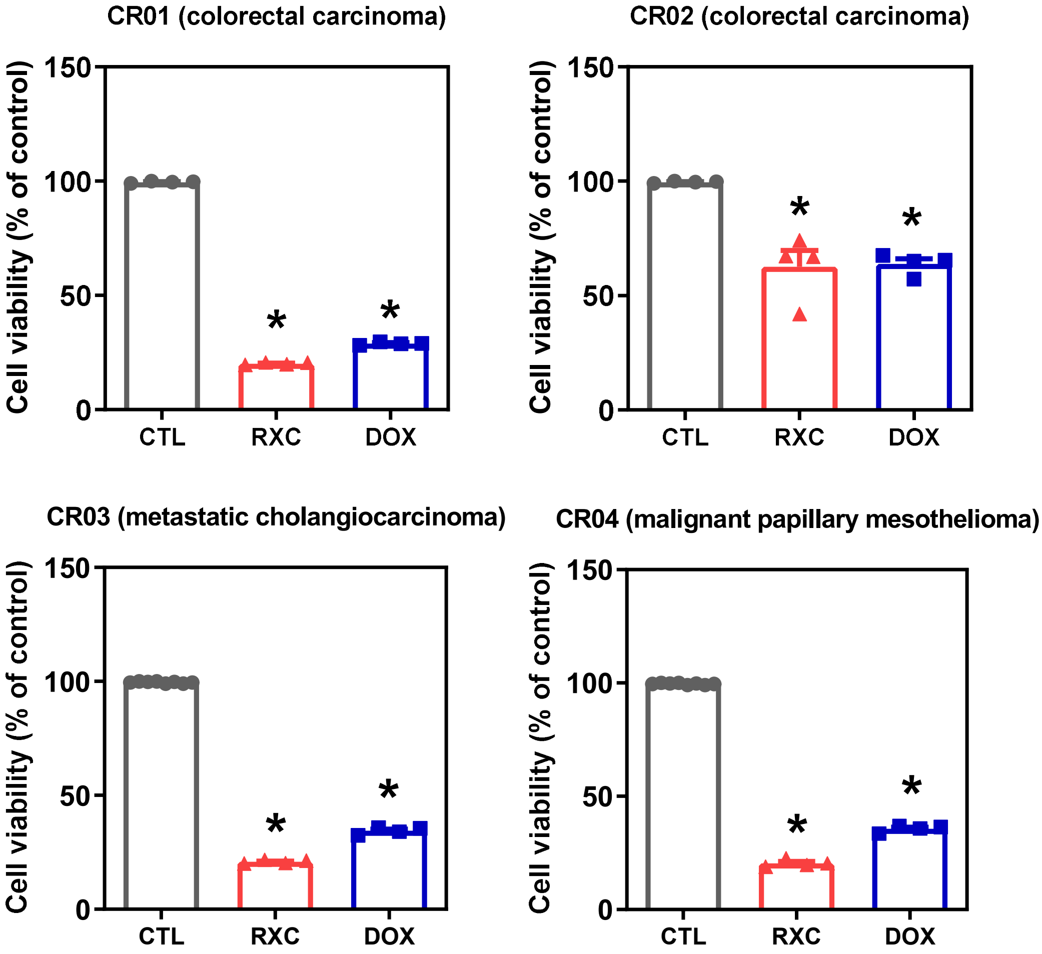

Figure S1. The effect of RXC on primary cancer cells. Cells were exposed to 25 μg/mL of each compound (RXC = 31.3 μM, DOX = 46 μM), and cell viability was measured after 96 h using the Alamar blue assay. The data are presented as the mean ± S.E.M. of four replicates. * $P < 0.05$ compared to CTL by one-way ANOVA followed by Dunnett's multiple comparisons test.

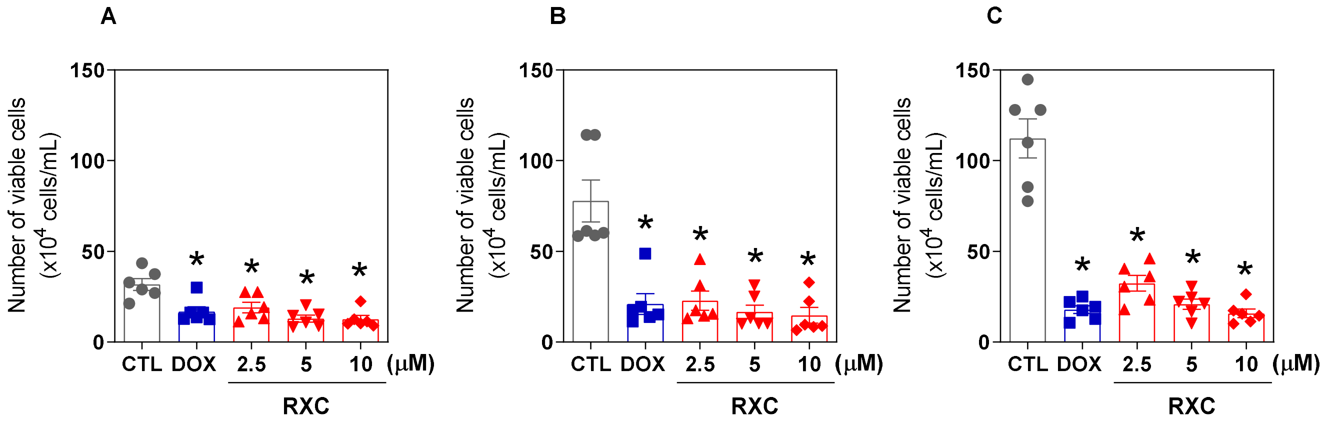

Figure S2. Trypan blue exclusion assay after (**A**) 24, (**B**) 48 and (**C**) 72 h of treatment with RXC in HCT116 cells. Vehicle (0.2% DMSO) was used as a negative control (CTL), and doxorubicin (DOX, 1 μM) was used as a positive control. Data are expressed as the mean ± S.E.M. of at least three repetitions (done in duplicate). * $P < 0.05$ compared with CTL by one-way ANOVA followed by Dunnett's multiple comparisons test.

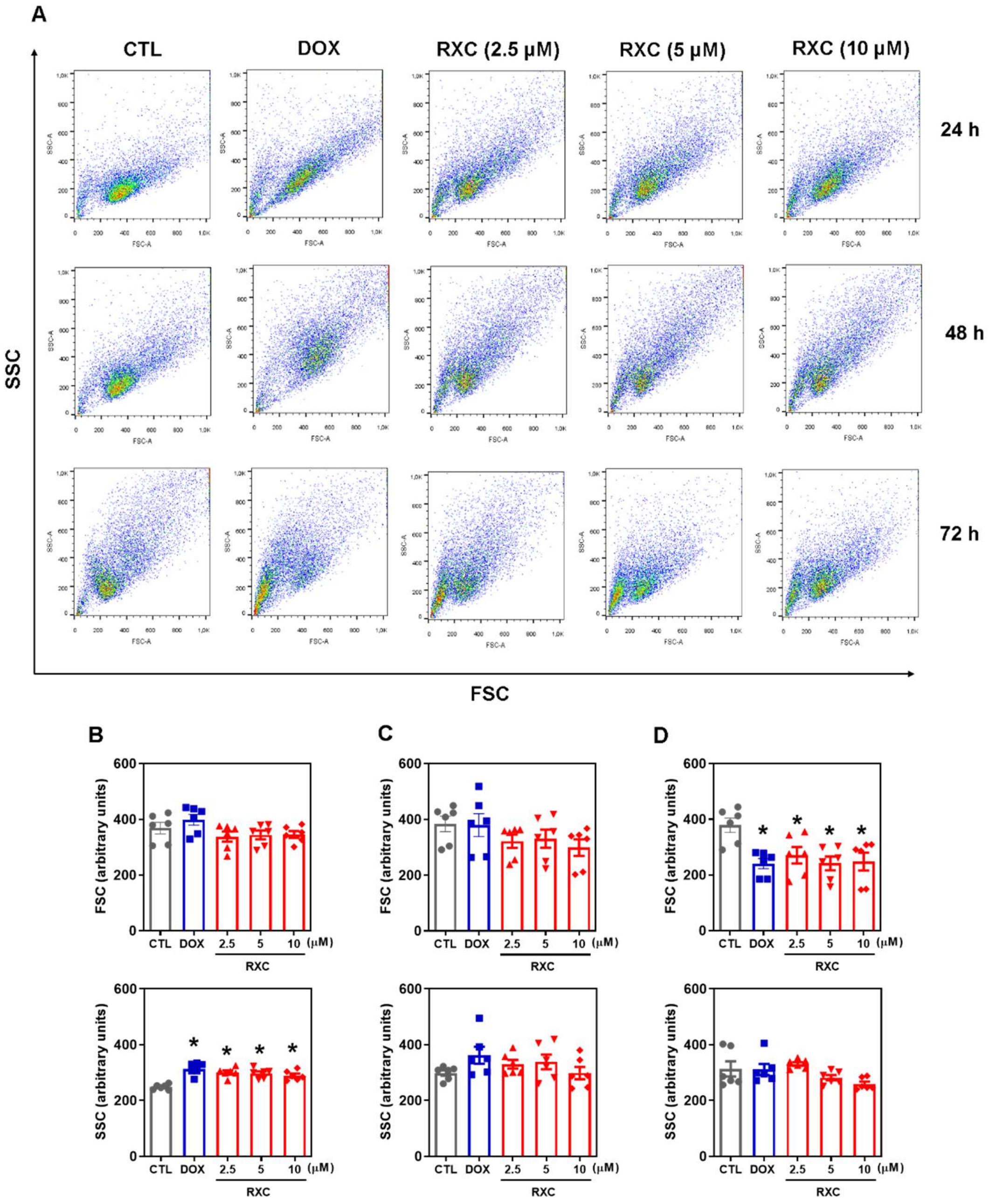

Figure S3. Effect of RXC on the morphology of HCT116 cells, as determined by light-scattering features detected by flow cytometry after 24 (**A** and **C**), 48 (**A** and **C**) and 72 (**A** and **D**) h of incubation. The vehicle (0.2% DMSO) was used as a control (CTL), and doxorubicin (DOX, 1 µM) was used as a positive control. Data are shown as the mean ± S.E.M. of at least three repetitions (done in duplicate). * $P < 0.05$ compared to CTL by one-way ANOVA followed by Dunnett's multiple comparisons test. FSC = forward scatter. SSC = side scatter.

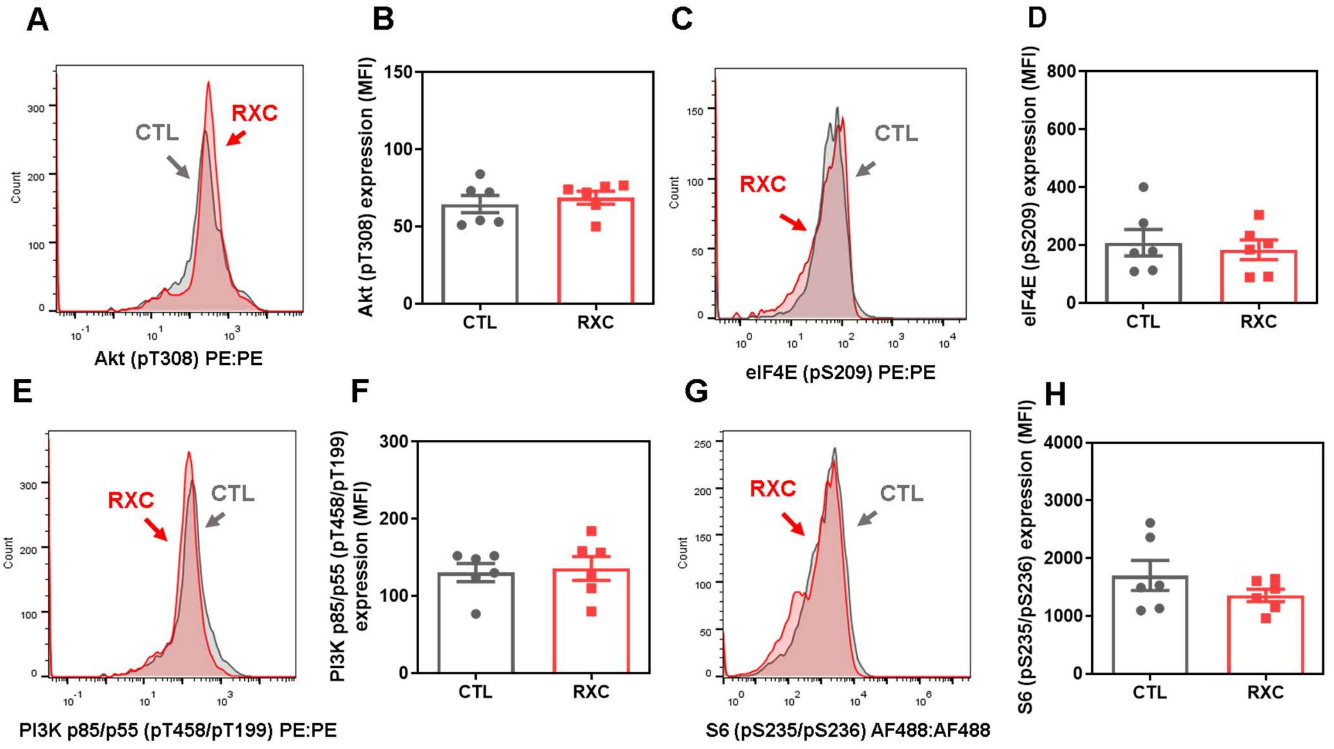

Figure S4. Quantification of Akt (pT308) (**A** and **B**), eIF4E (pS209) (**C** and **D**), PI3K p85/p55 (pT458/pT199) (**E** and **F**) and S6 (pS234/pS236) (**G** and **H**) expression in HCT116 cells after 24 h of incubation with 10 μM RXC, as determined by flow cytometric analysis. The vehicle (0.2% DMSO) was used as a control (CTL). Data are shown as the mean ± S.E.M. of at least three repetitions (done in duplicate). MFI: Mean fluorescence intensity.

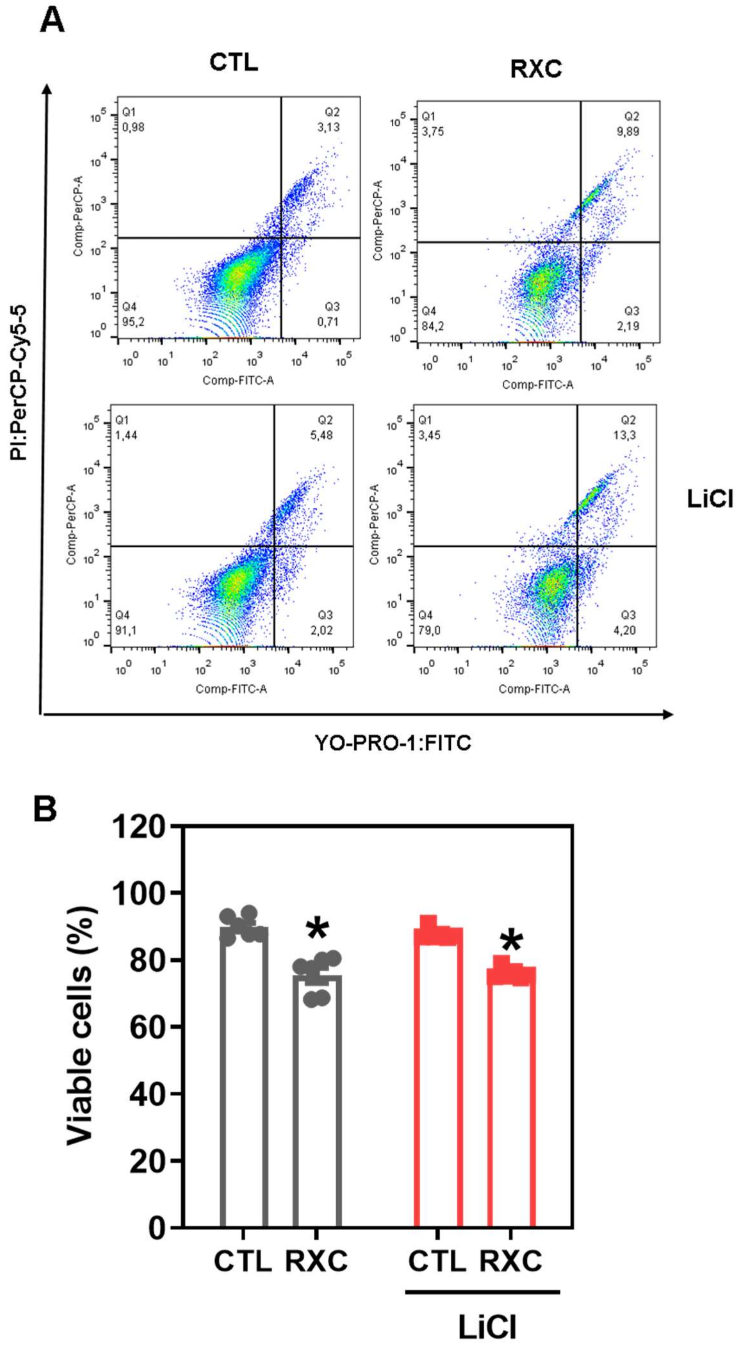

Figure S5. Action of lithium chloride (LiCl, a Wnt activator) on RXC-induced HCT116 cell death. (**A**) Representative flow cytometric dot plots. (**B**) Quantification of viable HCT116 cells (YO-PRO-1/PI double-negative cells). The cells were pretreated with 25 mM LiCl and then incubated with 10 μM RXC for 48 h. The vehicle (0.2% DMSO) was used as a control (CTL). Data are shown as the mean ± S.E.M. of at least three repetitions (done in duplicate). * $P < 0.05$ compared to CTL by Student's *t* test.

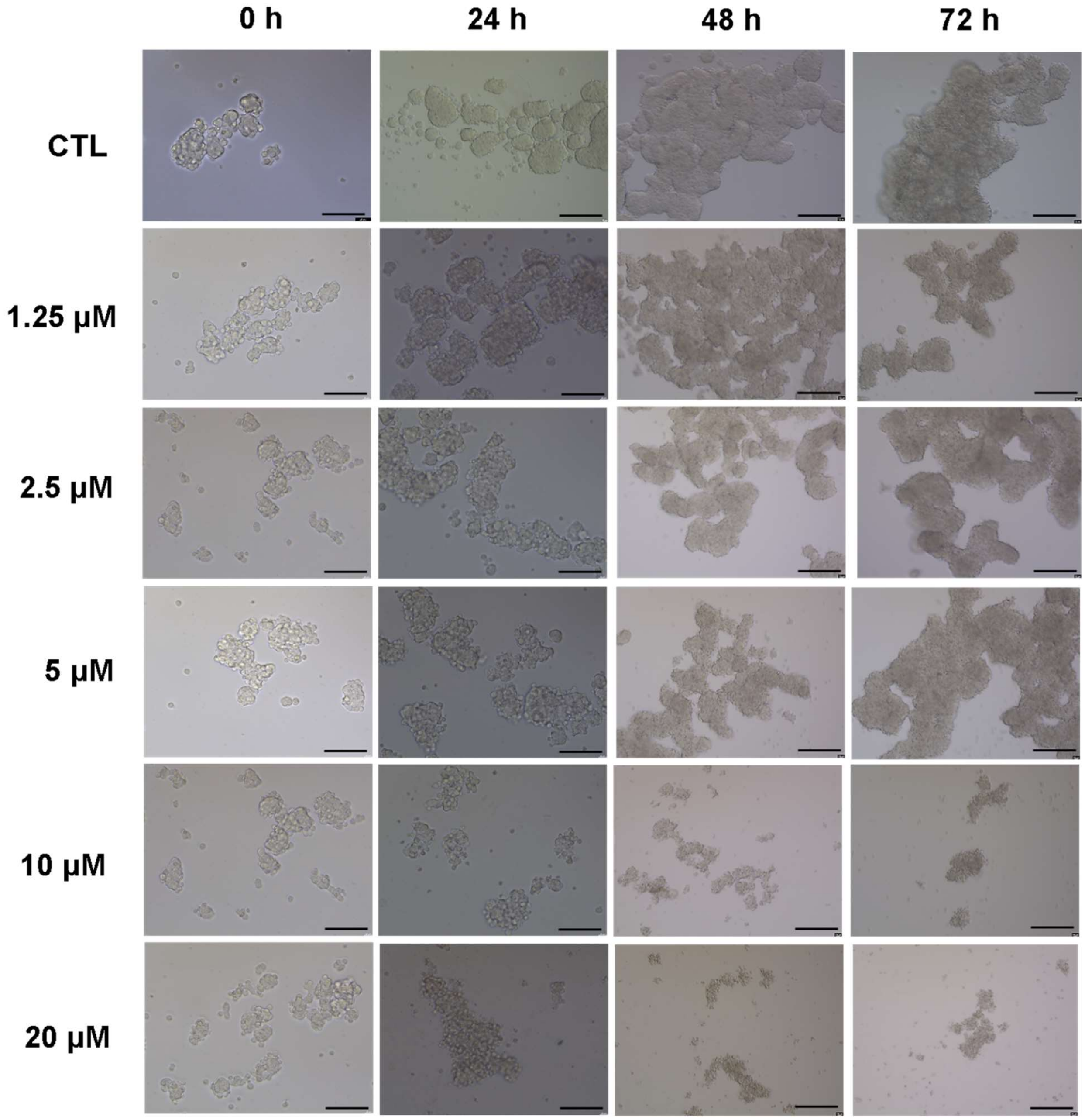

Figure S6. Representative images of colonospheres of HCT116 cells. The vehicle (0.2% DMSO) was used as a control (CTL). Scale bar = 100 µm.

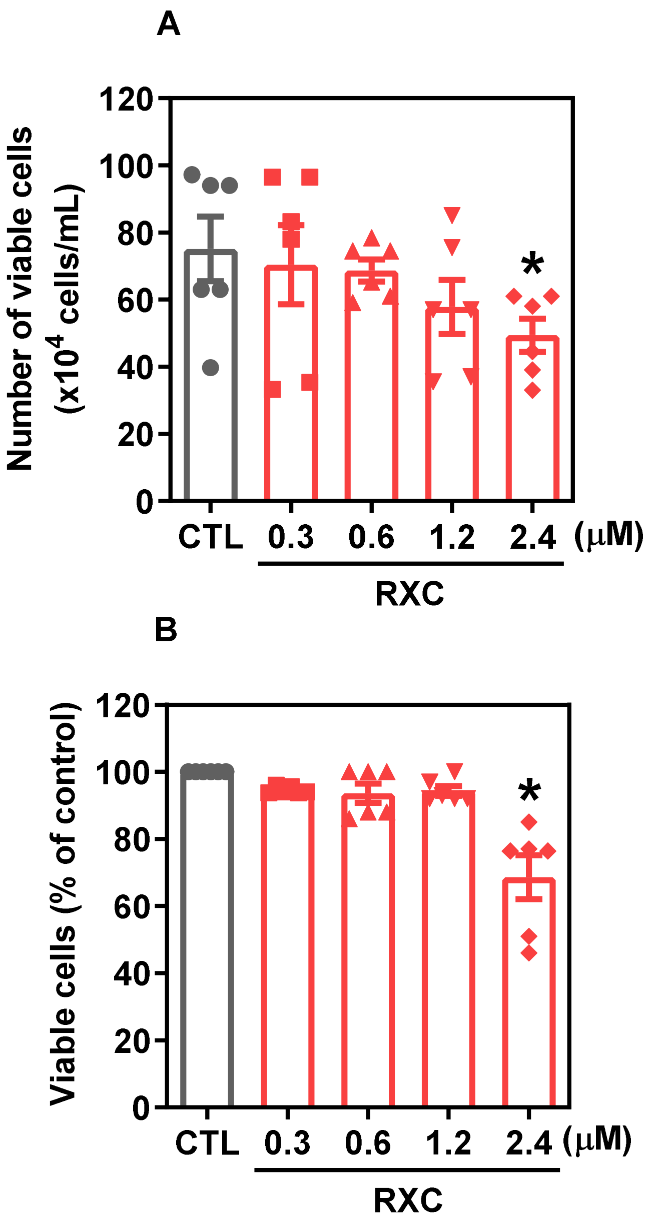

Figure S7. Quantification of viable HCT116 cells after 72 h incubation with RXC, as determined by trypan blue exclusion assay (**A**) and Alamar blue assay (**B**). The vehicle (0.2% DMSO) was used as a control (CTL). Data are shown as the mean ± S.E.M. of at least three repetitions (done in duplicate). * $P < 0.05$ compared to CTL by one-way ANOVA followed by Dunnett's multiple comparisons test.

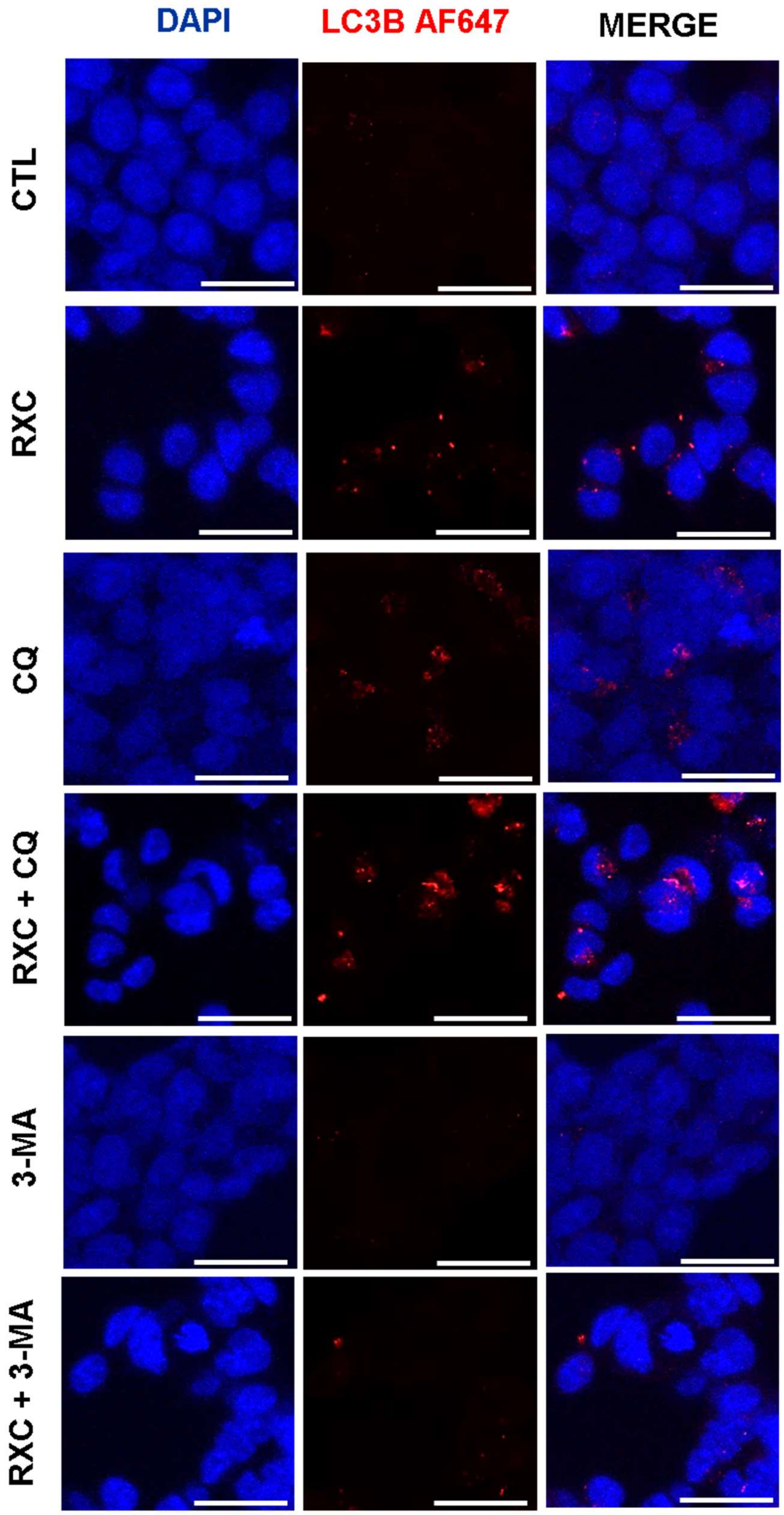

Figure S8. Representative immunofluorescence images of LC3B in HCT116 cells. The cells were pretreated with 50 μM CQ or 5 mM 3-MA and then incubated with 10 μM RXC for 24 h. The vehicle (0.2% DMSO) was used as a control (CTL). Scale bar = 25 μm.

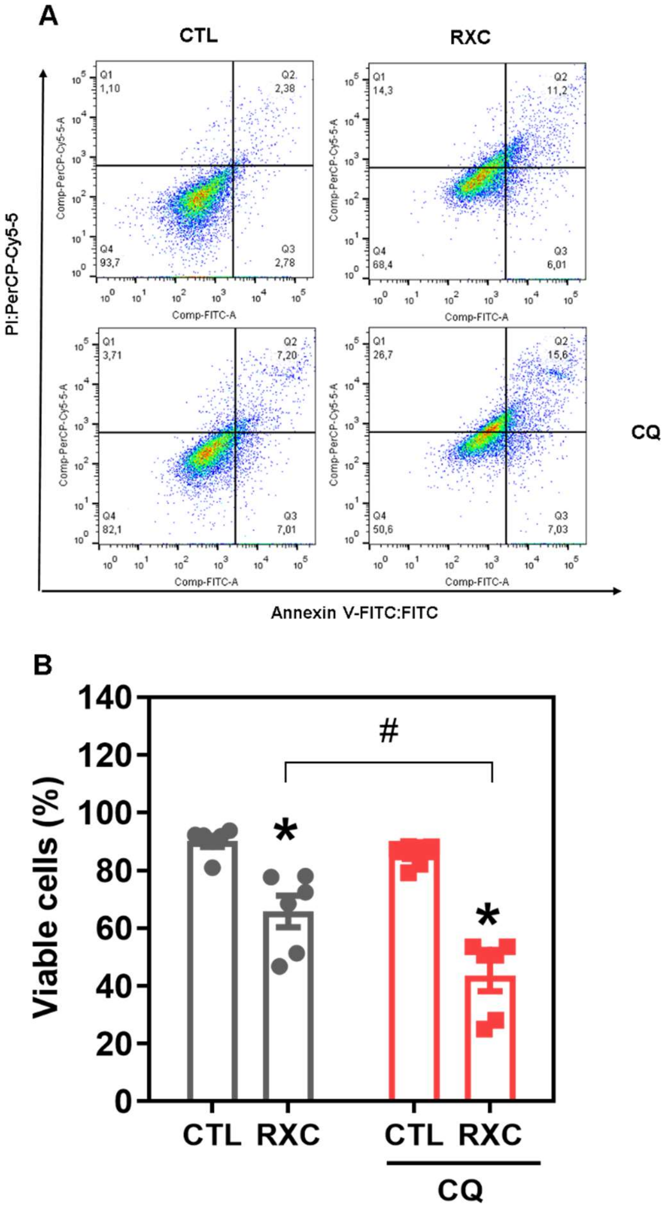

Figure S9. Effect of chloroquine (CQ, an autophagy inhibitor) on RXC-induced cell death in HCT116 cells. (**A**) Representative flow cytometric dot plots. (**B**) Quantification of viable HCT116 cells (annexin V-FITC/PI double-negative cells). The cells were pretreated with 50 μM CQ and then incubated with 10 μM RXC for 48 h. The vehicle (0.2% DMSO) was used as a control (CTL). Data are shown as the mean ± S.E.M. of at least three repetitions (done in duplicate). * $P < 0.05$ compared to CTL by Student's *t* test. # $P < 0.05$ compared to the respective treatment without inhibitor by Student's *t* test.

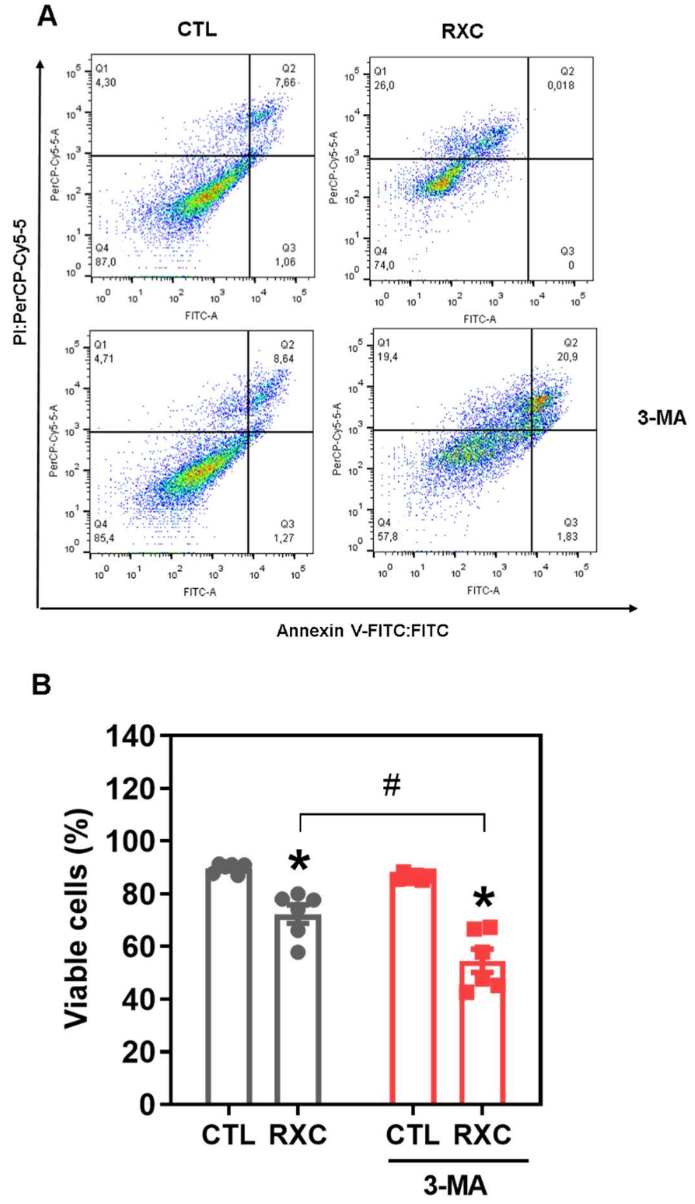

Figure S10. Effect of 3-methyladenine (3-MA, an autophagy inhibitor) on RXC-induced cell death in HCT116 cells. (**A**) Representative flow cytometric dot plots. (**B**) Quantification of viable HCT116 cells (annexin V-FITC/PI double-negative cells). The cells were pretreated with 5 mM 3-MA and then incubated with 10 μM RXC for 48 h. The vehicle (0.2% DMSO) was used as a control (CTL). Data are shown as the mean ± S.E.M. of at least three repetitions (done in duplicate). * $P < 0.05$ compared to CTL by Student's *t* test. # $P < 0.05$ compared to the respective treatment without inhibitor by Student's *t* test.

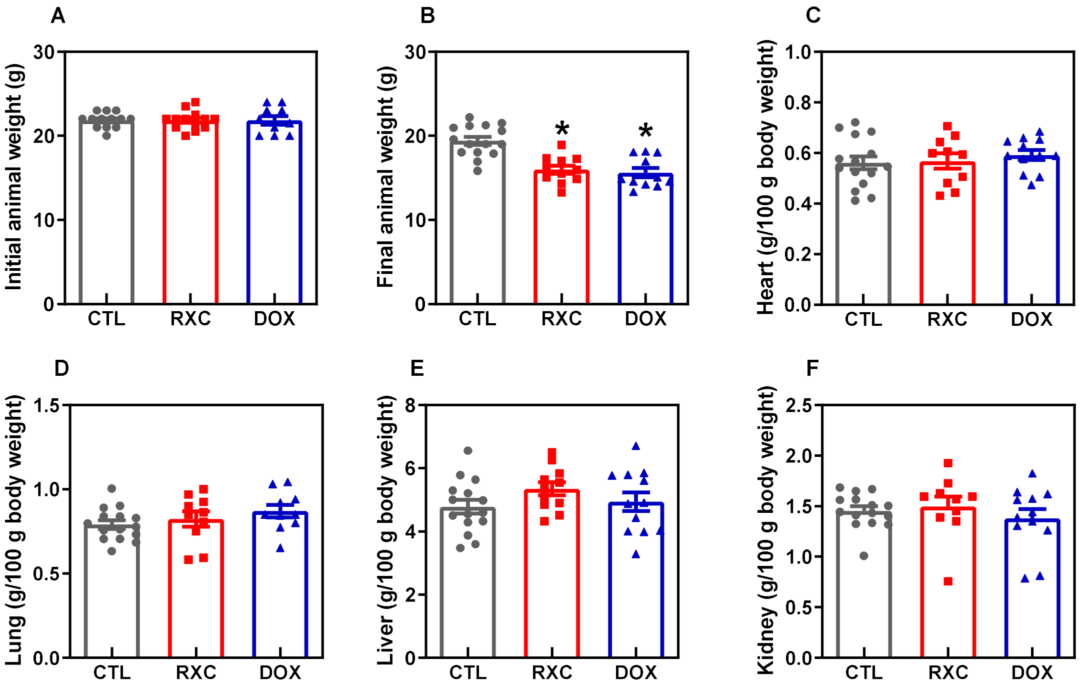

Figure S11. Effect of RXC treatment (2 mg/kg) on the body weight and relative organ weight of C. B-17 SCID mice inoculated with HCT116 cells. The negative control (CTL) was treated with the vehicle (5% DMSO) used to dilute RXC. DOX (0.8 mg/kg) was used as a positive control. Data are presented as the mean ± S.E.M. from 11-15 animals. * *P* < 0.05 compared to CTL by one-way ANOVA followed by Dunnett's multiple comparisons test.

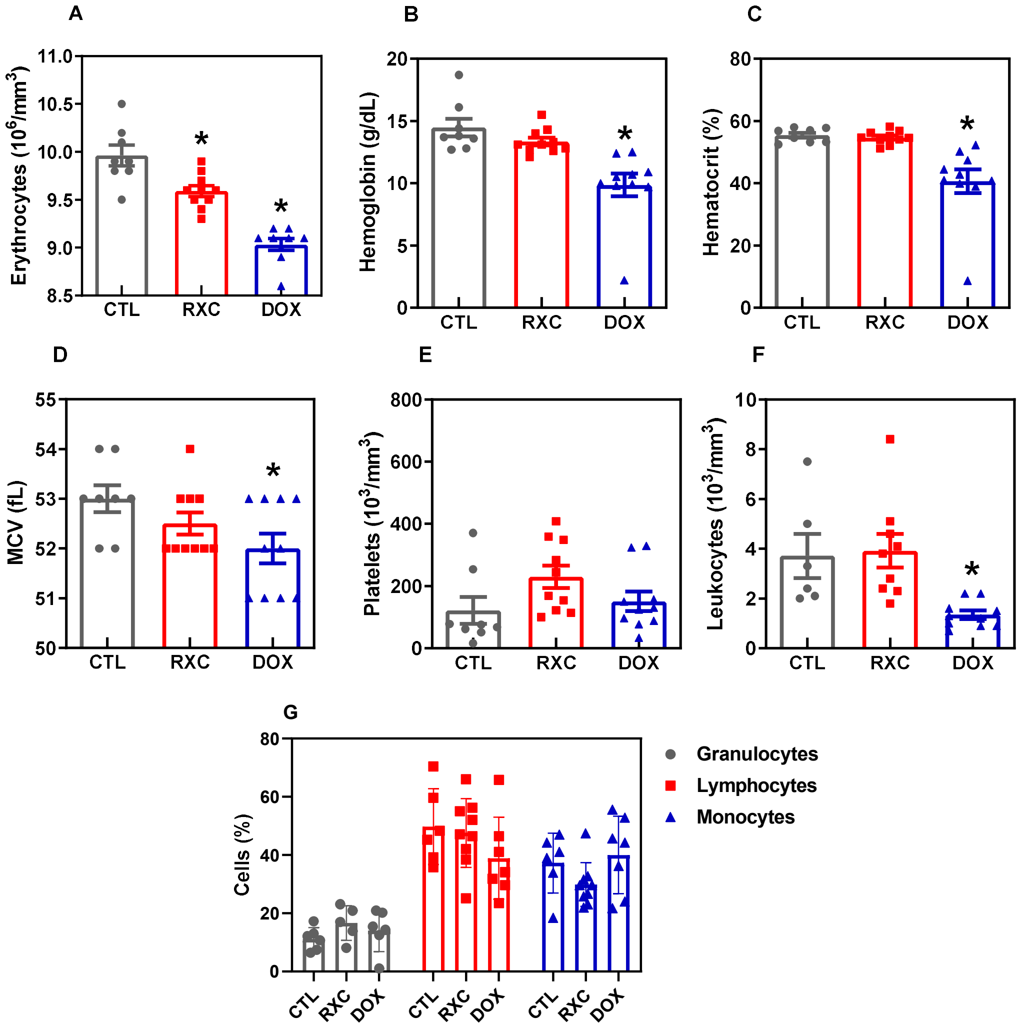

Figure S12. Effect of RXC treatment (2 mg/kg) on hematological parameters of peripheral blood of C. B-17 SCID mice inoculated with HCT116 cells. The negative control (CTL) was treated with the vehicle (5% DMSO) used to dilute RXC. Data are presented as the mean ± S.E.M. from 6-9 animals. * $P < 0.05$ compared to CTL by one-way ANOVA followed by Dunnett's multiple comparisons test.

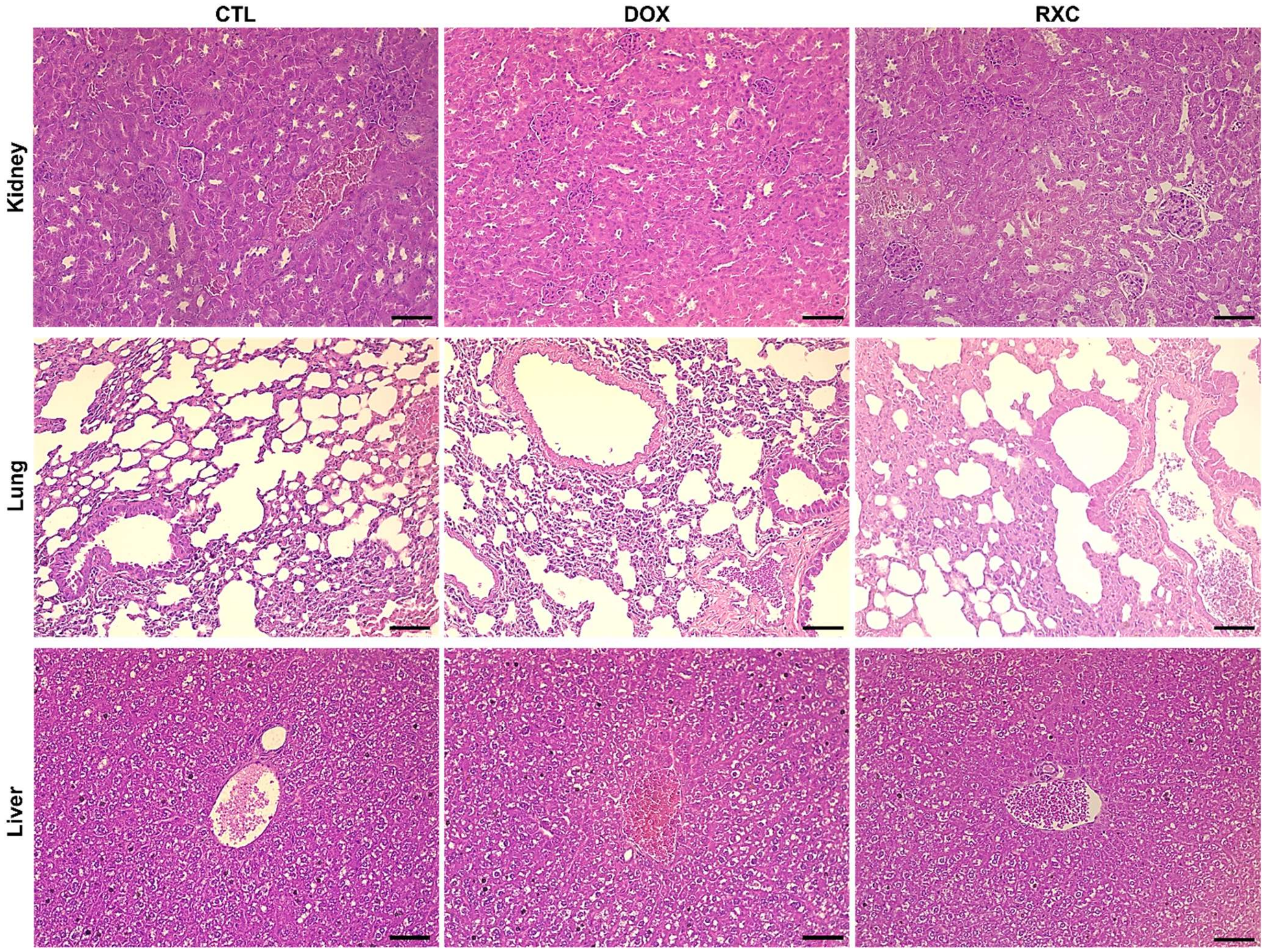

Figure S13. Representative histological analysis of the kidneys, lungs and livers of C. B-17 SCID mice inoculated with HCT116 cells and treated with RXC (2 mg/kg). The tissues were stained with hematoxylin and eosin and analyzed by light microscopy. The negative control (CTL) was treated with the vehicle (5% DMSO) used to dilute RXC. DOX (0.8 mg/kg) was used as a positive control. Scale bar = 50 µm.

Table S1. IC_{50} values against cancer and noncancerous cells

Cells	IC_{50} and 95%CI (in µM)	
	**RXC**	**DOX**
Cancer cells		
HCT116	6.5	3.3
	5.01 – 7.88	2.36 – 4.41
HepG2	6.28	0.09
	4.01 – 6.31	0.07 – 0.12
NB4	3.38	0.11
	1.25 – 8.19	0.02 – 0.45
THP-1	2.80	0.12
	1.78 - 4.39	0.07 - 0.20
JUKART	4.08	0.03
	2.11 – 7.23	0.01 – 0.14
K-562	3.88	1.50
	1.76 – 8.49	0.49 – 4.45
HL-60	3.2	0.09
	2.17 – 4.72	0.03 – 0.16
KG-1a	2.31	0.4
	1.63 – 3.26	0.2 – 0.6
MDA-MB-231	2.36	0.55
	1.68 – 3.31	0.23 – 1.26
MCF-7	9.55	1.76
	5.44 – 16.76	1.06 – 2.94
4T1	9.10	1.70
	4.98 – 16.61	1.17 – 2.46
HSC-3	4.84	1.43
	3.35 – 6.99	0.91 – 2.20
CAL 27	5.4	0.18
	2.58 – 11.29	0.05 – 0.62
SCC-25	10.23	1.58
	5.58 – 18.71	0.86 – 2.75

SCC4	1.89<br>0.81 – 4.39	0.07<br>0.02 – 0.23
SCC-9	2.81<br>1.9 – 4.16	1.21<br>0.71 – 1.89
A549	7.03<br>5.00 – 9.87	2.35<br>1.95 – 2.83
H1299	10.90<br>6.92 – 17.18	0.77<br>0.54 – 1.08
PANC-1	11.83<br>7.55 – 18.55	1.06<br>0.69 – 1.6
OVCAR-3	1.47<br>1.01 – 2.15	1.01<br>0.62 – 1.65
DU 145	4.17<br>3.11 – 5.58	0.22<br>0.14 – 0.33
U-87 MG	7.03<br>4.2 – 11.8	0.44<br>0.27 – 0.68
A-375	3.46<br>2.75 – 4.37	0.16<br>0.12 – 0.2
B16-F10	2.58<br>1.23 – 5.41	0.53<br>0.40 – 0.71
Noncancerous cells		
MRC-5	21.45<br>16.11 – 28.55	1.61<br>0.55 – 4.63
BJ	0.76<br>0.38 – 1.55	0.73<br>0.31 – 1.69
PBMC	4.93<br>2.95 – 8.21	1.28<br>0.91 – 1.82

The IC_{50} values were obtained by nonlinear regression from three independent experiments performed in duplicate by the Alamar blue assay after 72 h of incubation. Doxorubicin (DOX) was used as a positive control.

Table S2. Selective indexes found

Cancer cells	Noncancerous cells					
	MRC-5		BJ		PBMC	
	RXC	DOX	RXC	DOX	RXC	DOX
HCT116	3.3	0.5	0.3	0.2	1.2	0.4
HepG2	3.4	0.3	0.1	0.1	0.8	14.2
NB4	6.4	14.6	0.5	6.6	2.4	11.6
THP-1	7.7	13.4	0.6	6.1	2.9	10.7
JUKART	5.3	53.7	0.4	24.3	2.0	42.7
K-562	5.5	1.1	0.4	0.5	2.1	0.9
HL-60	6.7	17.9	0.2	8.1	1.5	14.2
KG-1a	9.3	4.0	0.3	1.8	2.1	3.2
MDA-MB-231	9.1	2.9	0.3	1.3	2.1	2.3
MCF-7	2.3	0.9	0.2	0.4	0.9	0.7
4T1	2.4	1.0	0.2	0.4	0.9	0.8
HSC-3	4.4	1.1	0.3	0.5	1.7	0.9
CAL 27	4.0	8.9	0.3	4.1	1.5	7.1
SCC-25	2.1	1.0	0.2	0.5	0.8	0.8
SCC4	11.4	23.0	0.9	10.4	4.3	18.3
SCC-9	7.6	1.3	0.6	0.6	2.9	1.1
A549	3.1	0.7	0.1	0.3	0.7	0.5
H1299	2.0	2.1	0.1	1.0	0.5	1.7
PANC-1	1.8	1.5	0.1	0.7	0.4	1.2
OVCAR-3	14.6	1.6	0.5	0.7	3.4	1.3
DU 145	5.1	7.3	0.2	3.3	1.2	5.8
U-87 MG	3.1	3.7	0.1	1.7	0.7	2.9
A-375	6.2	10.1	0.2	4.6	1.4	8.0
B16-F10	6.2	3.0	0.6	1.4	3.1	2.4

Data were calculated using the following formula: SI = IC_{50} [noncancerous cells]/IC_{50} [cancer cells].

Table S3. The effect of RXC on gene expression in HCT116 cells

Function/Assay ID	Gene symbol	Full name	RQ	
			CTL	RXC
**Apoptosis**				
Hs00608023_m1	BCL2	BCL2, apoptosis regulator	1.0	0.944
Hs04194392_s1	BIRC5	baculoviral IAP repeat containing 5	1.0	0.499
**PI3 Kinases & Phosphatases**				
Hs00234508_m1	MTOR	mechanistic target of rapamycin	1.0	0.595
Hs00904054_m1	PIK3C2A	phosphatidylinositol-4-phosphate 3-kinase catalytic subunit type 2 alpha	1.0	1.177
Hs00176908_m1	PIK3C3	phosphatidylinositol 3-kinase catalytic subunit type 3	1.0	1.485
Hs00907957_m1	PIK3CA	phosphatidylinositol-4,5-bisphosphate 3-kinase catalytic subunit alpha	1.0	0.956
**Growth Factors & Receptors**				
Hs01076090_m1	EGFR	epidermal growth factor receptor	1.0	1.135
Hs01001580_m1	ERBB2	erb-b2 receptor tyrosine kinase 2	1.0	0.312
Hs00176538_m1	ERBB3	erb-b2 receptor tyrosine kinase 3	1.0	0.814
Hs01128657_m1	FIGF	c-fos induced growth factor	N.d.	N.d.
Hs01052961_m1	FLT1	fms related tyrosine kinase 1	N.d.	N.d.
Hs01047677_m1	FLT4	fms related tyrosine kinase 4	1.0	3.841
Hs01547656_m1	IGF1	insulin like growth factor 1	N.d.	N.d.
Hs00609566_m1	IGF1R	insulin like growth factor 1 receptor	1.0	1.146
Hs04188276_m1	IGF2	insulin like growth factor 2		
Hs00911700_m1	KDR	kinase insert domain receptor	1.0	0.896
Hs00174029_m1	KIT	KIT proto-oncogene receptor tyrosine kinase	N.d.	N.d.

Hs00998018_m1	PDGFRA	platelet derived growth factor receptor alpha	N.d.	N.d.
Hs01019589_m1	PDGFRB	platelet derived growth factor receptor beta	1.0	2.249
**Drug Metabolism**				
Hs01561483_m1	ABCC1	ATP binding cassette subfamily C member 1	1.0	0.777
Hs00943350_g1	GSTP1	glutathione S-transferase pi 1	1.0	0.609
Hs00153133_m1	PTGS2	prostaglandin-endoperoxide synthase 2	1.0	1.461
Hs01555214_g1	TXN	thioredoxin	1.0	1.184
Hs00917067_m1	TXNRD1	thioredoxin reductase 1	1.0	3.038
**G-Protein Signaling**				
Hs00357608_m1	RHOA	ras homolog family member A	1.0	0.937
Hs03676562_s1	RHOB	ras homolog family member B	1.0	1.762
**Hormone Receptors**				
Hs01046816_m1	ESR1	estrogen receptor 1	1.0	1.523
Hs01100353_m1	ESR2	estrogen receptor 2	1.0	1.673
Hs01556702_m1	PGR	progesterone receptor	N.d.	N.d.
**Heat Shock Proteins**				
Hs00743767_sH	HSP90AA1	heat shock protein 90 alpha family class A member 1	1.0	0.353
Hs00427665_g1	HSP90B1	heat shock protein 90 beta family member 1	1.0	0.285
**Receptor Tyrosine Kinase Signaling**				
Hs00178289_m1	AKT1	AKT serine/threonine kinase 1	1.0	0.624
Hs01086099_m1	AKT2	AKT serine/threonine kinase 2	1.0	0.896
Hs00157817_m1	GRB2	growth factor receptor bound protein 2	1.0	1.220
**Cathepsins**				
Hs00947439_m1	CTSB	cathepsin B	1.0	1.307
Hs00157205_m1	CTSD	cathepsin D	1.0	0.826

Hs00964650_m1	CTSL	cathepsin L	1.0	1.073
Hs00175407_m1	CTSS	cathepsin S	1.0	0.500
**Cell Cycle**				
Hs00947994_m1	CDC25A	cell division cycle 25A	1.0	1.174
Hs00938777_m1	CDK1	cyclin dependent kinase 1	1.0	1.021
Hs01548894_m1	CDK2	cyclin dependent kinase 2	1.0	1.057
Hs00364847_m1	CDK4	cyclin dependent kinase 4	1.0	1.065
Hs00358991_g1	CDK5	cyclin dependent kinase 5	1.0	0.446
Hs00361486_m1	CDK7	cyclin dependent kinase 7	1.0	1.621
Hs00992501_g1	CDK8	cyclin dependent kinase 8	1.0	0.943
Hs00977896_g1	CDK9	cyclin dependent kinase 9	1.0	0.986
Hs00540450_s1	MDM2	MDM2 proto-oncogene	1.0	2.327
Hs00967238_m1	MDM4	MDM4, p53 regulator	1.0	1.960
Hs00972650_m1	TERT	telomerase reverse transcriptase	1.0	0.866
**Topoisomerases, Type II**				
Hs01032137_m1	TOP2A	topoisomerase (DNA) II alpha	1.0	0.769
Hs00172259_m1	TOP2B	topoisomerase (DNA) II beta	1.0	1.055
**Transcription Factors**				
Hs01095345_m1	ATF2	activating transcription factor 2	1.0	0.856
Hs00153153_m1	HIF1A	hypoxia inducible factor 1 alpha subunit	1.0	0.663
Hs00158114_m1	IRF5	interferon regulatory factor 5	1.0	1.472
Hs00765730_m1	NFKB1	nuclear factor kappa B subunit 1	1.0	1.055
Hs01034249_m1	TP53	tumor protein p53	1.0	1.058
**Protein Kinases**				
Hs01582072_m1	AURKA	aurora kinase A	1.0	0.343
Hs00945858_g1	AURKB	aurora kinase B	1.0	0.562
Hs00152930_m1	AURKC	aurora kinase C	1.0	0.498
Hs00983227_m1	PLK1	polo like kinase 1	1.0	0.890
Hs00198320_m1	PLK2	polo like kinase 2	1.0	1.023
Hs00177725_m1	PLK3	polo like kinase 3	1.0	1.749

Hs00179514_m1	PLK4	polo like kinase 4	1.0	1.081
Hs00925200_m1	PRKCA	protein kinase C alpha	1.0	0.567
Hs00176998_m1	PRKCB	protein kinase C beta	1.0	0.993
Hs01090047_m1	PRKCD	protein kinase C delta	1.0	1.557
Hs00942886_m1	PRKCE	protein kinase C epsilon	1.0	1.461
**RAS Signaling**				
Hs00978050_g1	HRAS	HRas proto-oncogene, GTPase	1.0	1.867
Hs00364284_g1	KRAS	KRas proto-oncogene, GTPase	1.0	2.847
Hs00180035_m1	NRAS	NRas proto-oncogene, GTPase	1.0	0.893
**Histone Deacetylases**				
Hs00978031_g1	HDAC1	histone deacetylase 1	1.0	0.509
Hs00978031_g1	HDAC11	histone deacetylase 11	1.0	1.433
Hs00231032_m1	HDAC2	histone deacetylase 2	1.0	0.509
Hs00187320_m1	HDAC3	histone deacetylase 3	1.0	0.879
Hs01041648_m1	HDAC4	histone deacetylase 4	1.0	1.233
Hs00997427_m1	HDAC6	histone deacetylase 6	1.0	0.969
Hs01045864_m1	HDAC7	histone deacetylase 7	1.0	0.975
Hs00954353_g1	HDAC8	histone deacetylase 8	1.0	0.826
**Poly ADP-Ribose Polymerases**				
Hs00242302_m1	PARP1	poly(ADP-ribose) polymerase 1	1.0	0.485
Hs00173105_m1	PARP4	poly(ADP-ribose) polymerase family member 4	1.0	0.791
Hs00186671_m1	TNKS	tankyrase	1.0	2.951
**Structural Proteins**				
Hs00362403_g1	NTN3	netrin 3	1.0	2.364

HCT116 cells were treated with 10 μM RXC for 12 h. The negative control (CTL) was treated with the vehicle (0.2% DMSO) used to dilute the compound tested. After treatment, total RNA was isolated and reverse transcribed. Gene expression was detected using a TaqMan® Array Human Cancer Drug Targets

96-well plate. The HPRT1, TFRC and YWHAZ genes were used as endogenous genes for normalization. Values represent the relative quantitation (RQ) compared with the calibrator (cells treated with the negative control). The genes were considered to be upregulated if RQ ≥ 2 and downregulated if RQ ≤ 0.5. N.d. Not detected (indicates no gene expression).

Table S4. List of cells used

Cells	Histological type	Species	Source[a,b,c]
Cancer cell lines			
HCT116	colorectal carcinoma	human	ATCC
HepG2	hepatocellular carcinoma	human	ATCC
NB4	acute promyelocytic leukemia	human	ATCC
THP-1	monocytic leukemia	human	ATCC
JUKART	T-cell lymphoid leukemia	human	ATCC
K-562	chronic myelogenous leukemia	human	ATCC
HL-60	acute promyelocytic leukemia	human	ATCC
KG-1a	acute myeloid leukemia	human	ATCC
MDA-MB-231	breast carcinoma	human	BCRJ
MCF-7	breast adenocarcinoma	human	ATCC
4T1	breast carcinoma	mouse	ATCC
HSC-3	oral squamous cell carcinoma	human	ATCC
CAL 27	oral squamous cell carcinoma	human	ATCC
SCC-25	oral squamous cell carcinoma	human	ATCC
SCC4	oral squamous cell carcinoma	human	ATCC
SCC-9	oral squamous cell carcinoma	human	ATCC
A549	lung adenocarcinoma	human	BCRJ
H1299	non-small cell lung carcinoma	human	BCRJ
PANC-1	pancreas ductal adenocarcinoma	human	BCRJ
OVCAR-3	ovarian carcinoma	human	BCRJ
DU 145	prostate carcinoma	human	BCRJ
U-87 MG	glioblastoma	human	BCRJ
A-375	melanoma	human	BCRJ
B16-F10	melanoma	mouse	ATCC
Noncancerous cell lines			
MRC-5	lung fibroblast	human	ATCC
BJ	foreskin fibroblast	human	ATCC
Mutant and parental cell lines			

BAD KO SV40 MEF	immortalized mouse embryonic fibroblasts with the BAD gene knocked out	mouse	ATCC
WT SV40 MEF	wild-type immortalized embryonic fibroblasts	mouse	ATCC
Primary cells			
CR01	colorectal carcinoma	human	primary cell culture
CR02	colorectal carcinoma	human	primary cell culture
CR03	metastatic cholangiocarcinoma	human	primary cell culture
CR04	malignant papillary mesothelioma	human	primary cell culture
PBMC	heath peripheral blood mononuclear cells	human	primary cell culture

[a]ATCC denotes American Type Culture Collection (U.S. and BCRJ denotes the Rio de Janeiro Cell Bank (Brazil). [b]Primary cell cultures of CR01, CR02, CR03 and CR04 were obtained by enzymatic digestion of tumor fragments using trypsin (0.25%) and kept in a water bath at 37°C for 100 min. The dissociated fragments were filtered through a 100 µm cell strainer (BD Biosciences, USA). Cells were centrifuged, washed and resuspended in RPMI 1640 or DMEM-F12 medium containing 20% FBS and 1% antibiotic. Cells were plated at 5 x 10^5 cells/well. The Research Ethics Committee of São Rafael Hospital (Salvador, Bahia, Brazil) approved the protocol (CAAE 30815214.9.3002.0048). [c]Primary cell culture of PBMCs was obtained from peripheral blood from healthy donors by a standard Ficoll density protocol. Then, PBMCs were resuspended in RPMI 1640 or DMEM-F12 medium with 20% FBS and 1% antibiotic. Cells were plated at 5 x 10^5 cells/well. Concanavalin A (10 µg/mL, Sigma–Aldrich) was used as a mitogen to trigger cell division in T lymphocytes and was added at the beginning of the culture. The Research Ethics Committee of the Oswaldo Cruz Foundation (Salvador, Bahia, Brazil) approved the protocol (CAAE 16220713.2.0000.0040).

Table S5. List of antibodies used

Epitope	Fluorchrome	Clone	Application	Catalog number	Company
4EBP1 (pT36/pT45)	AF488	M31-16	Intracellular staining	560287	BD Phosflow
Akt (pS473)	AF488	M89-61	Intracellular staining	560404	BD Phosflow
Akt (pT308)	PE	J1-223.371	Intracellular staining	558275	BD Phosflow
Akt 1	PE	55/PKBa/Akt	Intracellular staining	560049	BD Phosflow
CD133	PE	W6B3C1 (W6B3)	Cell surface staining	566594	BD Pharmingen
Cleaved PARP (Asp214)	PE	F21-852	Intracellular staining	552933	BD Pharmingen
E-Cadherin	BV421	36/E-Cadherin	Cell surface staining	564186	BD Horizon
elF4E (pS209)	PE	J77-925	Intracellular staining	560229	BD Phosflow
GSK3 (pS9)	AF488	D85E12	Intracellular staining	14026S	Cell Signaling Technology
HSP90	PE	C45G5	Intracellular staining	70657S	Cell Signaling Technology
LC3B	AF647	1251A	Intracellular staining	IC9390R	R&D Systems
mTOR (pS2448)	PE	O21-404	Intracellular staining	563489	BD Phosflow
NF-κB p65 (pS529)	AF488	K10-895.12.50	Intracellular staining	558421	BD Phosflow
p62/SQSTM1	AF488	864807	Intracellular staining	IC8028G	R&D Systems

PI3K p85/p55 (pT458/pT199)	PE	PI3KY458-1A11	Intracellular staining	MAS-28027	Invitrogen
S6 (pS235/pS236)	AF488	N7-548	Intracellular staining	560434	BD Phosflow
Vimentin	AF488	RV202	Intracellular staining	562338	BD Pharmingen
IgG1, κ Isotype Control	PE	MOPC-21	Cell surface staining	556650	BD Pharmingen
